# Berberine alleviates liver fibrosis through inducing ferrous redox to activate ROS-mediated hepatic stellate cells ferroptosis

**DOI:** 10.1038/s41420-021-00768-7

**Published:** 2021-12-04

**Authors:** Jiazhi Yi, Shuyun Wu, Siwei Tan, Yunfei Qin, Xing Wang, Jie Jiang, Huiling Liu, Bin Wu

**Affiliations:** 1 Department of Gastroenterology, The Third Affiliated Hospital of Sun Yat-Sen University, 510630 Guangzhou, China; 2 Guangdong Provincial Key Laboratory of Liver Disease Research, 510630 Guangzhou, China; 3 Department of The Biological Therapy Center, The Third Affiliated Hospital of Sun Yat-Sen University, 510630 Guangzhou, China

**Keywords:** Stress signalling, Macroautophagy

## Abstract

Berberine (BBR) has been explored as a potential anti-liver fibrosis agent, but the underlying mechanisms are unknown. In the current study, we aimed to investigate the molecular mechanisms underlying the effect of BBR against liver fibrogenesis in thioacetamide (TAA) and carbon tetrachloride (CCl_4_) induced mouse liver fibrosis. In addition to i.p. injection with TAA or CCl_4_, mice in the treatment group received BBR intragastrically. Concurrently, combined with TAA and BBR treatment, mice in the inhibitor group were injected i.p. with ferrostatin-1 (Fer-1). Hepatic stellate cells (HSCs) were also used in the study. Our results showed that BBR obviously alleviated mouse liver fibrosis and restored mouse liver function; however, the pharmacological effects of BBR against liver fibrosis were significantly diminished by Fer-1 treatment. Mechanically, BBR impaired the autophagy–lysosome pathway (ALP) and increased cell reactive oxygen species (ROS) production in HSCs. ROS accelerated the breakdown of the iron-storage protein ferritin and sped up iron release from ferritin, which resulted in redox-active iron accumulation in HSCs. Lipid peroxidation and glutathione (GSH) depletion triggered by the Fenton reaction promoted ferroptosis and attenuated liver fibrosis. Furthermore, impaired autophagy enhanced BBR-mediated ferritin proteolysis to increase cellular ferrous overload via the ubiquitin–proteasome pathway (UPS) in HSCs and triggered HSC ferroptosis. Collectively, BBR alleviated liver fibrosis by inducing ferrous redox to activate ROS-mediated HSC ferroptosis. Our findings may be exploited clinically to provide a potential novel therapeutic strategy for liver fibrosis.

## Introduction

Liver fibrosis is a growing global health problem characterized by activated hepatic stellate cells (HSCs) and excess deposition of fibrillary collagen [1]. Without effective intervention, it would likely progress to cirrhosis, liver failure, and even hepatocellular carcinoma [2]. Regulation of the activation and targeted scavenging of HSCs requires new targets for treatment and to reverse fibrogenesis; however, there is currently a lack of effective methods. Our previous work showed that targeted deletion of *ARRB1* in mice alleviated liver fibrosis by modulating autophagy [3]; other effects of dysfunctional HSC cells, including induction of apoptosis [4], lipocyte phenotype [5], and glycolytic function [6], could mitigate the pathological morphological changes in liver fibrosis.

As the major bioactive compound of Rhizoma Coptidis (Chinese name Huanglian), berberine (BBR) was found to be an effective therapeutic drug in many diseases, including inflammatory diseases [7], diabetes [8], and cancers [9]. Recently, BBR was found to inhibit HSC proliferation and extracellular matrix (ECM) production, alleviating liver fibrosis [10]. However, despite the positive outcomes, its underlying mechanism has not been elucidated.

Reactive oxygen species (ROS) are critical for maintaining many cellular processes, and the generation and scavenging of ROS needs to be precisely regulated [11]. Autophagy is one of the most important mechanisms in regulating ROS [12]. As a fundamental cellular process, autophagy plays a critical role in eliminating sources of ROS in response to diverse stress conditions and in maintaining the dynamic balance of ROS [13, 14]. Cells deficient in essential autophagy genes have increased basal ROS levels [15]. Impaired autophagy leads to increased oxidative stress and contributes to the production and accumulation of ROS, suggesting a possible amplification loop where oxidative stress amplifies ROS generation [16]. Therefore, ROS reduction by augmented autophagy is expected to contribute to the maintenance of cell stability.

As a form of regulated cell death (RCD), ferroptosis has become an emerging focus in many diseases [17]. Notably, iron metabolism plays an essential role in regulating vulnerability to ferroptosis [18]. Iron exists in two isoforms, ferric (Fe^3+^) and ferrous (Fe^2+^) ions, which are important components involved in multiple cellular processes [19]. Under the action of certain reductants, including superoxide (the major species of ROS), ferritin-bound Fe^3+^ can be reduced to Fe^2+^ and subsequently released from ferritin [20–22]. Then accumulated redox-active iron could redox cycle to produce a Fenton reaction and peroxidize lipids as well as the further disintegration of ferritin [21], promoting cell ferroptosis [23, 24].

The role of ferroptosis in liver fibrosis has not been clearly defined, and as a contradictory process [25, 26], it remains unknown whether ferroptosis is involved and acts as a central hub for berberine-mediated alleviation of liver fibrosis. In this study, we demonstrated that BBR inactivated HSCs and inhibited ECM production via ferroptosis in vitro and in vivo. Mechanistically, ferritin was modulated by BBR-mediated autophagy/ROS and the ubiquitin–proteasome system (UPS) pathway, undeniably leading to redox-active iron overload and iron homeostasis disruption, thus promoting the additional generation of ROS and inevitably triggering HSC ferroptosis.

## Results

### BBR alleviates liver fibrosis in mice

**Fig. 1:**
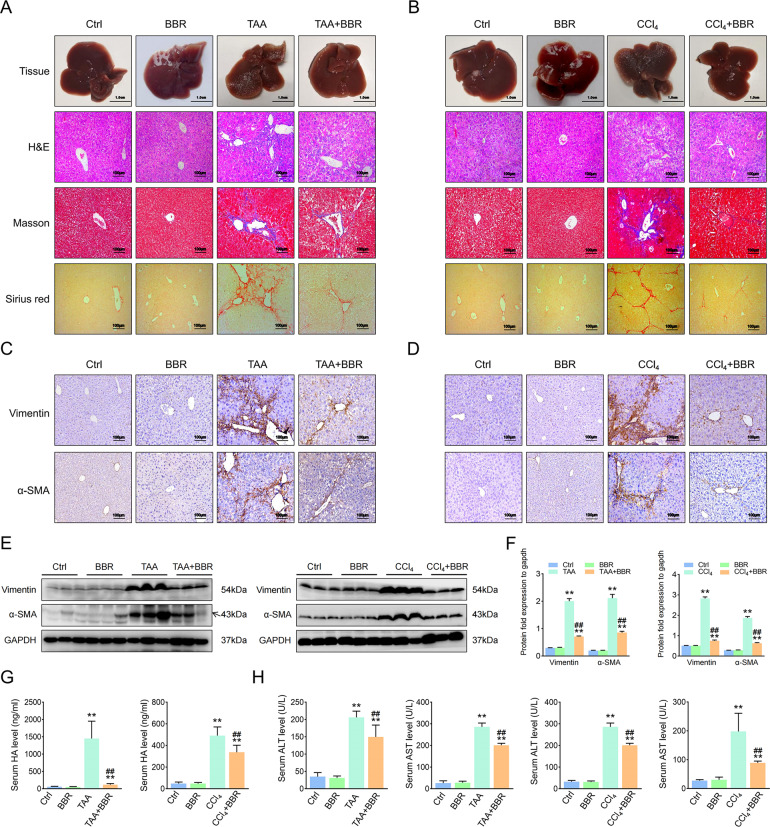
BBR alleviates liver fibrosis in mice. Mice were divided into: Control group (Ctrl, only i.p. with saline or oil, respectively), Berberine group (BBR, BBR 200 mg/kg/day intragastrically and i.p. with saline or oil, respectively), Liver fibrosis model group (TAA or CCl_4_, TAA/CCl_4_-induced hepatic fibrotic, i.p. with TAA or CCl_4_, respectively), and TAA- or CCl_4_-treated group plus BBR (TAA + BBR or CCl_4_ + BBR). **A**, **B** General examination of TAA/CCl_4_-induced mouse liver fibrosis by H&E, Masson’s trichrome, and Sirius red staining. **C**, **D** Vimentin and α-SMA are represented in TAA/CCl_4_-induced mouse liver fibrosis. **E**, **F** Protein expression of Vimentin and α-SMA in TAA/CCl_4_-induced mouse liver fibrosis. **G** Determination of serum hyaluronic acid. **H** Determination of serum ALT and AST. The results are expressed as mean ± SD. Data are representative of three independent experiments; *n* = 6 in every group; Scales: 1 cm or 100 μm. Representative photographs are shown. Compared with the control group, **P < 0.01; compared with the model group, ^##^*P* < 0.01.

We treated mice with BBR for 6 weeks, and their serum levels of ALT and AST were no higher than the control. In the mouse hepatic fibrosis models, liver fibrosis histopathological characteristics were presented, and BBR treatment obviously attenuated the mouse liver fibrosis (Fig. 1A, B), with downregulation of fibrosis markers and lower Ishak scores (Fig. 1C–F and Supplementary Fig. 1A–F). Then we checked serum levels of lactic dehydrogenase, hyaluronic acid, procollagen III, and collagen IV, and only hyaluronic acid was downregulated compared with the model group (Fig. 1G). Furthermore, BBR partially restored the abnormal serum levels of ALT and AST (Fig. 1H). Overall, these results indicate that BBR attenuates hepatic fibrosis in mice.

### BBR inhibits HSC activation in vivo and in vitro

**Fig. 2:**
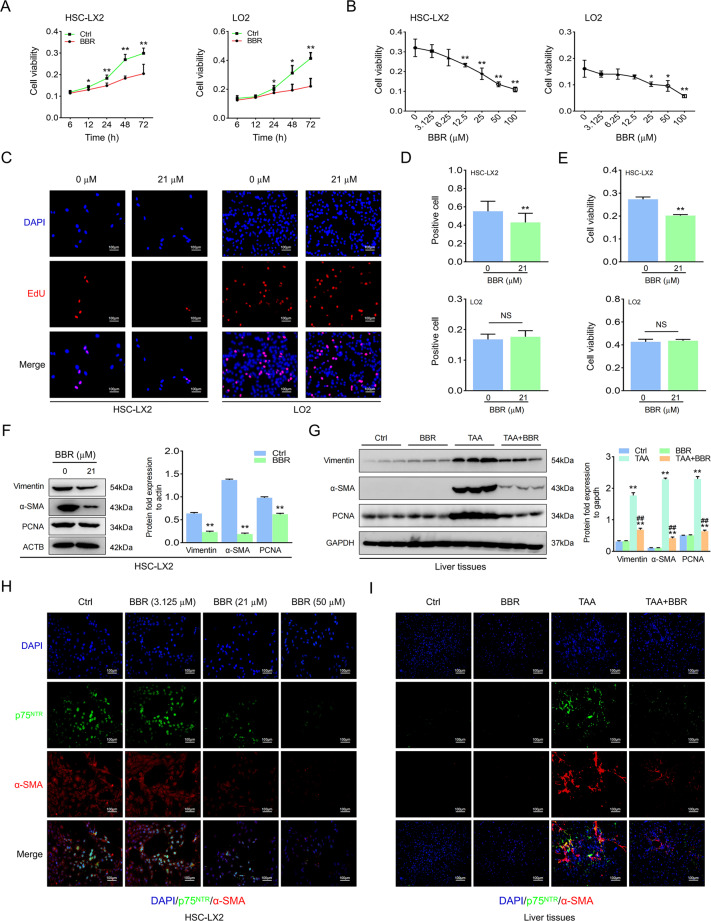
BBR inhibits HSC activation in vivo and in vitro. **A**, **B** HSC-LX2 and human hepatocyte LO2 cells were treated with BBR (50 μM) for the indicated time or cells were treated with different concentrations of BBR for 48 h, and cell viability was determined by CCK-8 assay. **C**–**E** Administration of HSC-LX2 and hepatocyte LO2 with BBR (21 μM) for 24 h; cell proliferation and viability were investigated by EdU and CCK-8 assays. **F** HSC-LX2 were treated with BBR (21 μM) for 24 h, then a whole-cell lysate was taken for western blot to investigate the Vimentin, α-SMA, and PCNA proteins. **G** Protein expression of Vimentin, α-SMA, and PCNA in TAA-induced mouse liver fibrosis. **H**, **I** Costaining of p75^NTR^ and α-SMA in HSC-LX2 or TAA-induced mouse hepatic fibrosis. The results are expressed as mean ± SD. Data are representative of three independent experiments; *n* = 3–6 in every group; Scales: 100 μm. Representative photographs are shown. Compared with the control group, *P < 0.05, **P < 0.01; compared with the model group, ^##^*P* < 0.01. NS, not significant.

HSC activation is the main cause of ECM synthesis in liver fibrosis, and we found that BBR dose- and time-dependently affected HSC viability and proliferation. Although BBR affected hepatocytes, it needed a longer time or a higher concentration compared to HSCs (Fig. 2A, B and Supplementary Fig. 2A, B). BBR at an IC50 concentration affected HSCs but not hepatocytes at the time indicated. Furthermore, it also downregulated protein expression (Fig. 2C–G and Supplementary Fig. 2C–G). p75^NTR^ is a marker of activated HSCs, and the loss of p75^NTR^ inhibits HSC activation [27]. Immunofluorescence (IF) staining indicated colocalization of alpha smooth muscle actin (α-SMA) with p75^NTR^ in the mouse model; however, upregulated p75^NTR^ could be reversed by BBR, together with downregulation of α-SMA, and similar results were verified in HSCs (Fig. 2H, I and Supplementary Fig. 2H, I). Above all, BBR attenuates hepatic fibrosis by inhibiting HSC activation in vivo and in vitro.

### Ferroptosis contributes to the inhibition of HSC activation

**Fig. 3:**
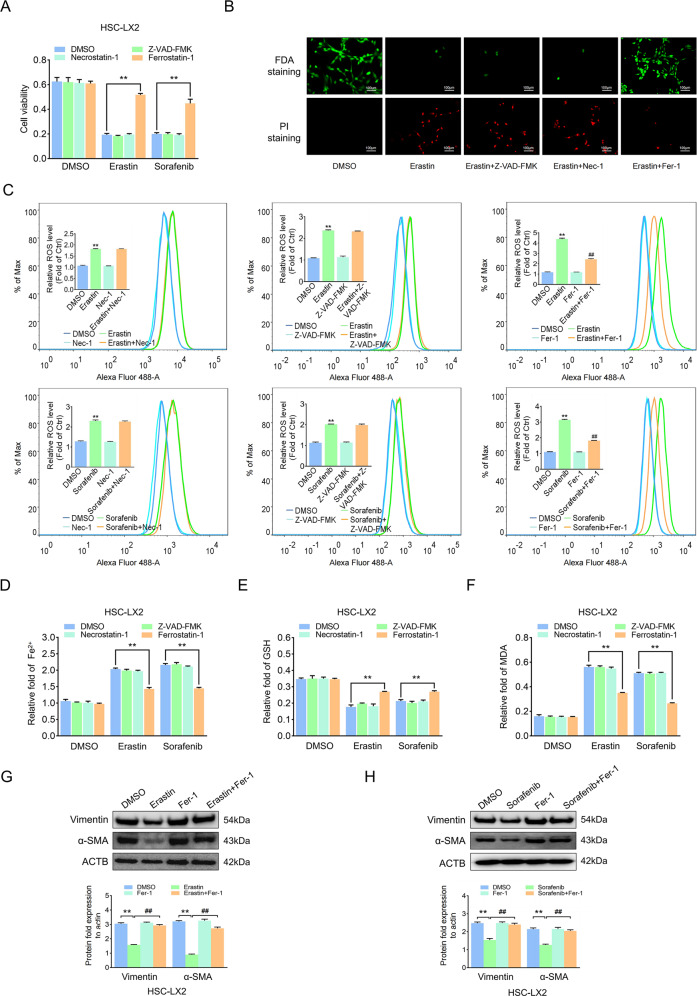
Ferroptosis contributes to the inhibition of HSC activation. HSC-LX2 cells were treated with erastin (10 μM) and sorafenib (10 μM) with or without the indicated inhibitors (Z-VAD-FMK, 10 μM; Fer-1, 1 μM; necrostatin-1, 10 μM) for 24 h. **A** Cell viability was assayed by CCK-8. **B** Live cells and dead cells were assayed by FDA staining and PI staining, respectively. **C**–**F** ROS generation, cellular Fe^2+^, GSH depletion, and lipid peroxidation MDA production was assayed. **G**, **H** Vimentin and α-SMA protein levels were assayed. The results are expressed as mean ± SD. Data are representative of three independent experiments; *n* = 3–6 in every group; Scales: 100 μm. Representative photographs are shown. Compared with the control group, **P < 0.01; compared with the ferroptosis-inducer group, ^##^*P* < 0.01.

Sorafenib and erastin can mediate ferroptosis in cancer cells, regarded as a new anticancer strategy [28], but how they function in benign diseases, including liver fibrosis, is unknown. We observed that erastin/sorafenib-induced growth inhibition in HSCs was blocked by the ferroptosis inhibitor Fer-1 but not the apoptosis inhibitor Z-VAD-FMK or the necroptosis inhibitor necrostatin-1 (Fig. 3A and Supplementary Fig. 3A). Fluorescein diacetate (FDA) and propidium iodide (PI) staining showed a drastic reduction in live cells and an obvious increase in cell death after exposure to erastin compared with the untreated group, and only Fer-1 abolished the promoting effect of erastin (Fig. 3B and Supplementary Fig. 3B). Redox-active iron accumulation, ROS generation, lipid peroxidation, and glutathione depletion are key events in ferroptosis [29]. Ferroptosis events were enhanced following erastin or sorafenib treatment; however, Fer-1 but not the other inhibitors could inhibit ferroptosis events (Fig. 3C–F and Supplementary Fig. 3C–F). Last, activation markers of HSCs were downregulated by erastin and sorafenib but Fer-1 treatment reversed this effect (Fig. 3G, H and Supplementary Fig. 3G, H). Overall, ferroptosis contributes to the inhibition of HSC activation in vitro.

### Autophagy is involved in BBR-induced depression of HSC activation in liver fibrosis

**Fig. 4:**
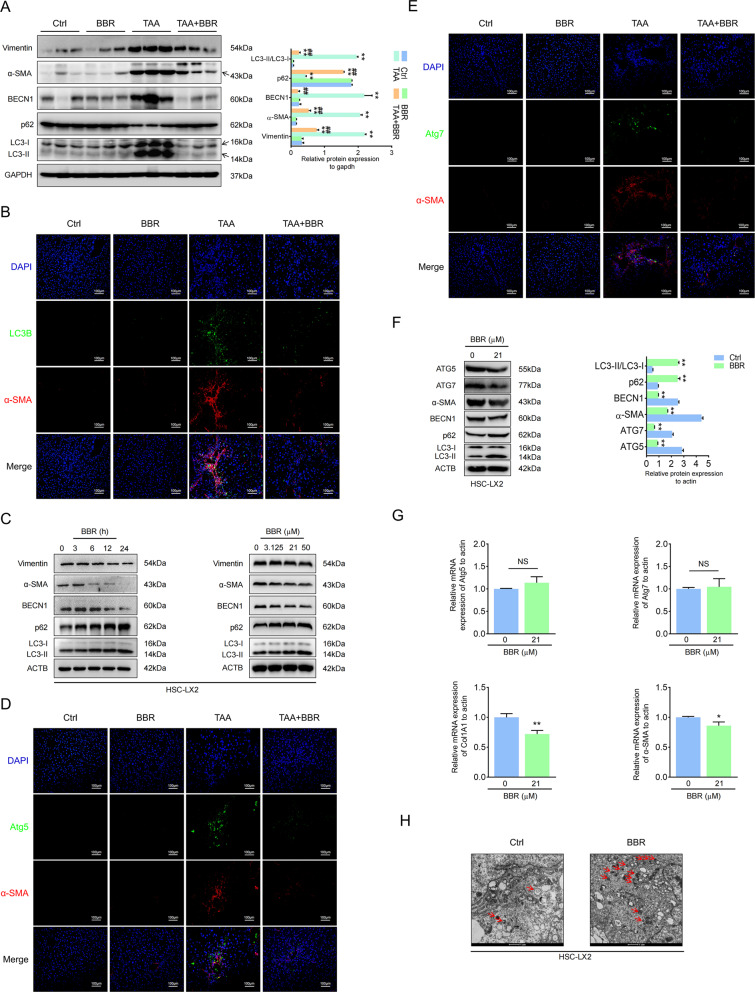
Autophagy is involved in BBR-induced depression of HSC activation in liver fibrosis. **A** The expression levels of BECN1, p62, LC3B, vimentin, and α-SMA were detected by western blot in TAA-induced mouse liver fibrosis. **B** Double IF staining of α-SMA and LC3B. **C** Western blot revealed that BBR inhibited autophagy in HSC-LX2. **D**, **E** Costaining of α-SMA and Atg5 or Atg7. **F**, **G** HSC-LX2 cells were treated with or without BBR (21 μM) for 24 h; protein expression of Atg5, Atg7, α-SMA, LC3B, and p62 and the relative mRNA levels of Atg5, Atg7, COL1A1, and α-SMA were assayed by western blot and qPCR. **H** HSC-LX2 cells were treated with or without BBR (21 μM) for 24 h; transmission electron microscopy ultrastructural features of autophagosomes are presented. The results are expressed as mean ± SD. Data are representative of three independent experiments; *n* = 3–6 in every group; Scales: 1 or 100 μm. Representative photographs are shown. Compared with the control group, *P < 0.05, **P < 0.01; compared with the model group, ^##^*P* < 0.01. NS, not significant.

Autophagy participates in many fibrotic diseases [3, 5, 30]. We noticed that autophagy was enhanced in mouse liver fibrosis and that BBR treatment could reverse it. BBR dose- and time-dependently impaired HSC autophagy, combined with a downregulation of activation markers (Fig. 4A, B and Supplementary Fig. 4A, B). In T6 cells, BBR inhibited autophagosome formation with a decrease in LC3B-II and an increase in p62 (Supplementary Fig. 4C, H), whereas in LX2 cells, we noticed an increasing number of autophagosomes with increasing expression of LC3B-II and p62 (Fig. 4C, H). Increased numbers of autophagosomes can be due to an increase in the formation of autophagosomes or a reduction in autophagosome turnover caused by late suppression of autophagy. We confirmed that BBR impaired autophagy by monitoring the process of autophagic flux in the presence of autophagy inhibitors chloroquine (CQ) and 3-methyladenine (3-MA) [31] in GFP-LC3B LX2 cells (Supplementary Fig. 5A–H). These results suggested that BBR impaired autophagic flux in both HSC-LX2 and T6 cells. Autophagy-related genes ATG5 and ATG7 play important roles in mediating autophagy [23]. BBR downregulated ATG5 and ATG7 expression with a decrease in α-SMA in HSCs, which is consistent with the double IF staining in mouse liver fibrosis. Quantitative real-time polymerase chain reaction (qPCR) revealed that BBR downregulated COL1A1 and α-SMA mRNA but not ATG5 and ATG7 mRNA (Fig. 4D–G and Supplementary Fig. 4D–G). This indicated that autophagy is involved in BBR-induced impairment of HSC activation in liver fibrosis.

### Suppression of autophagy enhances BBR-induced HSC ferroptosis

**Fig. 5:**
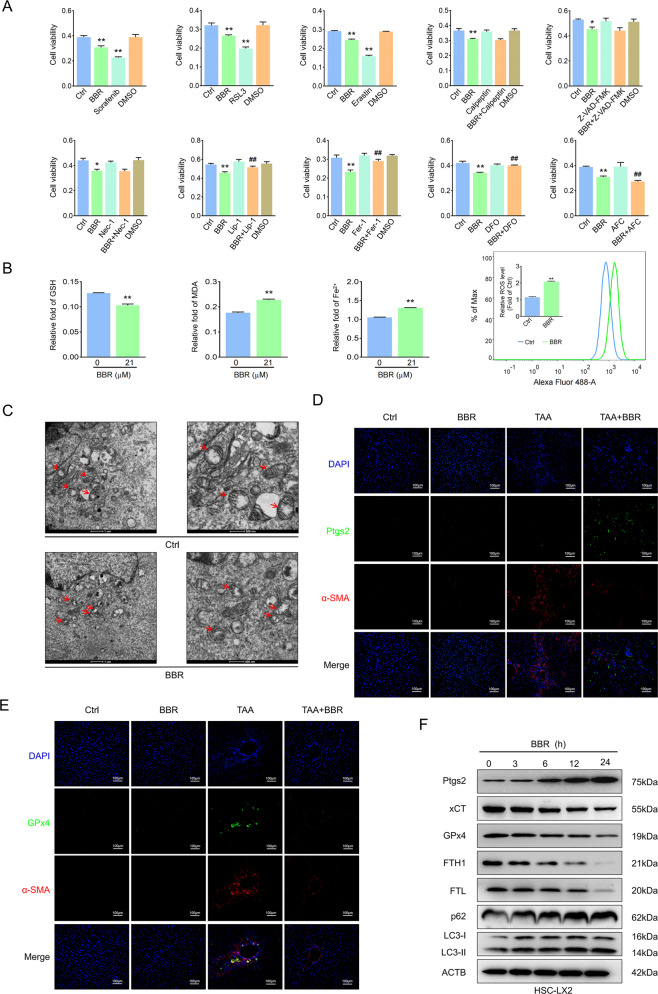
Suppression of autophagy enhances BBR-induced HSC ferroptosis. **A** HSC-LX2 cells were treated with BBR (21 μM), erastin (10 μM), RSL3 (1 μM), sorafenib (10 μM), or specific inhibitors of different cell death pathways (necrostatin-1 10 μM, Fer-1 1 μM, liproxstatin-1 100 nM, DFO 25 μM, Z-VAD-FMK 10 μM, and calpeptin 10 μM). Cell viability was assayed by CCK-8. **B** HSC-LX2 was exposed to BBR (21 μM) for 24 h; a whole-cell lysate was taken for cellular Fe^2+^, ROS production, MDA generation, and GSH depletion investigation. **C** HSC-LX2 were treated with or without BBR (21 μM) for 24 h; transmission electron microscopic ultrastructural features of mitochondria are shown. **D**, **E** Costained α-SMA with GPx4 or Ptgs2 in TAA-induced mouse liver fibrosis. **F** HSC-LX2 cells were treated with BBR (21 μM) for the indicated time, a whole-cell lysate was analyzed by western blot using antibodies against the indicated proteins. The results are expressed as mean ± SD. Data are representative of three independent experiments; *n* = 3–6 in every group; Scales: 1 μm, 100 μm, or 500 nm. Representative photographs are shown. Compared with the control group, *P < 0.05, **P < 0.01; compared with the BBR group, ^##^*P* < 0.01.

Blocking autophagy leads to inactivation of HSCs and attenuation of liver fibrosis [3]. We noticed a huge increase in the expression of the ferroptosis marker ferritin heavy chain 1 (FTH1) in both clinical samples and mouse liver fibrosis. Perls’-DAB staining showed a clearly ferric iron deposition mainly in the zone of fibrotic scar regions, which indirectly represent the cellular iron storage protein ferritin in activated HSCs, while BBR treatment decreased FTH1 deposition (Supplementary Fig. 6A–D). We hypothesized that BBR inhibited HSC autophagy to relieve hepatic fibrosis through degrading ferritin and promoting HSC ferroptosis. We found that either ferroptosis inducer or BBR obviously decreased HSC viability and resulted in cell death. This was worsened when combined with ammonium ferric citrate. However, ferroptosis inhibitors or the iron chelating agents deferoxamine or deferiprone but not other inhibitors inhibited the BBR-induced cell viability decrease (Fig. 5A and Supplementary Fig. 6E), and BBR treatment triggered ferroptotic events (Fig. 5B and Supplementary Fig. 6F). HSC morphological features of ferroptosis were evaluated by transmission electron microscopy (TEM). Compared with control cells, we found that dense and shrunken mitochondria were notably apparent in HSCs exposed to BBR (Fig. 5C and Supplementary Fig. 6G). Furthermore, double IF staining showed that the ferroptosis marker GPx4 accumulates in fibrotic livers and always flocks together around fibrotic scar regions with α-SMA. However, BBR treatment downregulated both GPx4 and α-SMA expression. Ptgs2, another marker of ferroptosis, which is rarely found around fibrotic scar regions where robust staining of α-SMA is present, was dramatically enhanced along with downregulation of α-SMA in BBR-treated fibrosis mice (Fig. 5D, E and Supplementary Fig. 6H). To further confirm ferroptosis was associated with impaired autophagy, double IF staining of LC3B with Ptgs2 or FTH1 revealed LC3B colocalization with Ptgs2 or FTH1 in fibrotic scar regions, BBR treatment markedly reduced the expression of LC3B and FTH1 and enhanced Ptgs2 expression, which was consistent with the western blot data (Fig. 5F and Supplementary Fig. 6I–K). In summary, these results validate that autophagy is involved in BBR-mediated HSC ferroptosis and suppression of autophagy enhanced BBR-induced HSC ferroptosis.

### BBR downregulates autophagy to enhance ROS/ferrous-mediated HSC ferroptosis

**Fig. 6:**
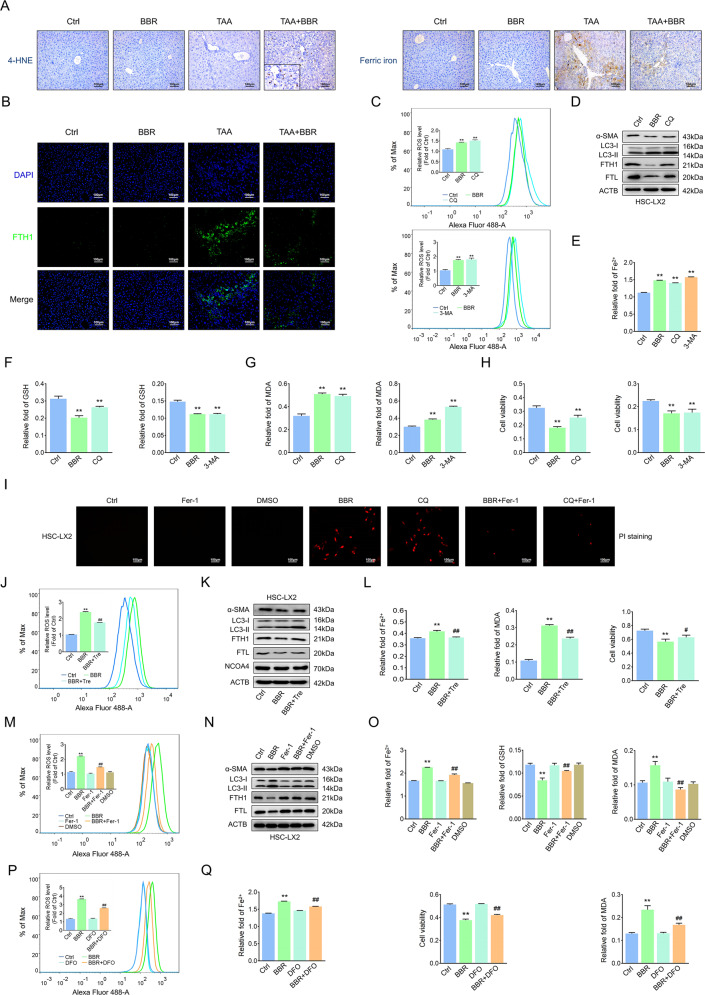
BBR downregulates autophagy to enhance ROS/ferrous-mediated HSC ferroptosis. **A**, **B** IHC for 4-HNE, Perls’-DAB for ferric ion and IF for FTH1 in TAA-induced hepatic fibrosis mouse. **C** HSC-LX2 was exposed to BBR (21 μM), CQ (10 μM) or 3-MA (5 mM) for 24 h; lipid ROS was investigated. **D** HSC-LX2 was treated with BBR (21 μM) or CQ (10 μM) for 24 h; markers of fibrosis, autophagy, and ferroptosis were assayed. **E**–**H** HSC-LX2 was exposed to BBR (21 μM), CQ (10 μM), or 3-MA (5 mM) for 24 h; cellular Fe^2+^, MDA, GSH, and cell viability were investigated. **I** HSC-LX2 cells were treated with BBR (21 μM) with or without CQ (10 μM) or Fer-1 (1 μM) for 24 h, and cell death was quantified by PI staining. **J**–**L** HSC-LX2 was exposed to BBR (21 μM) followed by trehalose (50 mM) for 12 h; lipid ROS, markers of autophagy, ferroptosis, cellular Fe^2+^, MDA, and cell viability were investigated. **M**–**O** HSC-LX2 cells were pretreated with Fer-1 (1 μM) for 1 h, followed by BBR (21 μM) for 24 h; lipid ROS, markers of autophagy, ferroptosis, cellular Fe^2+^, GSH, and MDA were investigated. **P**, **Q** HSC-LX2 was pretreated with DFO (25 µM) for 1 h, following treatment with BBR (21 μM) for 24 h; lipid ROS, cellular Fe^2+^, cell viability, and MDA were investigated. The results are expressed as mean ± SD. Data are representative of three independent experiments; *n* = 3–6 in every group; Scales: 100 μm. Representative photographs are shown. Compared with the control group, **P < 0.01; compared with the BBR group, ^#^*P* < 0.05, ^##^*P* < 0.01.

Impaired autophagy promotes the failure of protein and organelle quality control and increases basal levels of ROS, which could lead to increased oxidative stress [12, 13, 16]. Therefore, the reduction of ROS by augmented autophagy is expected to contribute to the maintenance of cell stability. We found that BBR treatment enhanced 4-HNE expression in fibrotic scar regions in mouse liver fibrosis. However, BBR reduced ferric iron deposition and decreased FTH1 expression, reflecting a relationship between ROS and ferritin (Fig. 6A, B and Supplementary Fig. 7A, B). Then impaired autophagy by BBR, CQ, or 3-MA markedly enhanced cell ROS levels and triggered ferroptosis events. Western blot analysis showed a downregulation of α-SMA and LC3-II, together with degradation of ferritin light chain (FTL) and FTH1. PI staining showed enhanced cell death, but this effect could be reversed by Fer-1 (Fig. 6C–I and Supplementary Fig. 7C–I). The autophagy-inducer trehalose obviously reversed BBR-mediated ferroptosis (Fig. 6J–L and Supplementary Fig. 7J–L). Interestingly, neither BBR nor trehalose impacted NCOA4 expression (Fig. 6K and Supplementary Fig. 7K), suggesting that NCOA4-mediated ferritinophagy was not involved in BBR-mediated ferritin degradation or trehalose-induced ferritin turnover [32]. Next, the lipid ROS scavenger Fer-1 blocked BBR-induced ferroptotic events (Fig. 6M–O and Supplementary Fig. 7M–O). Ferritin-bound Fe^3+^ can be reduced to Fe^2+^ and subsequently released from ferritin by the action of certain reductants, including superoxide [20–22], and the released ferrous iron could then redox cycle to produce ROS, including superoxide (O_2_^●−^) and hydroxyl radicals (^●^OH) as well as other superoxides and provoke membrane lipid peroxidation, which could then accelerate the disintegration of ferritin [21] and drive cells to ferroptosis [23, 24]. HSCs treated with deferoxamine or deferiprone significantly inhibited BBR-induced ferroptotic events and increased cell viability (Fig. 6P, Q and Supplementary Fig. 7P, Q). These data suggest that BBR alleviates liver fibrosis by downregulating autophagy to enhance ROS/ferrous-mediated HSC ferroptosis.

### BBR promotes ferritin proteolysis to increase ferrous overload in HSCs

**Fig. 7:**
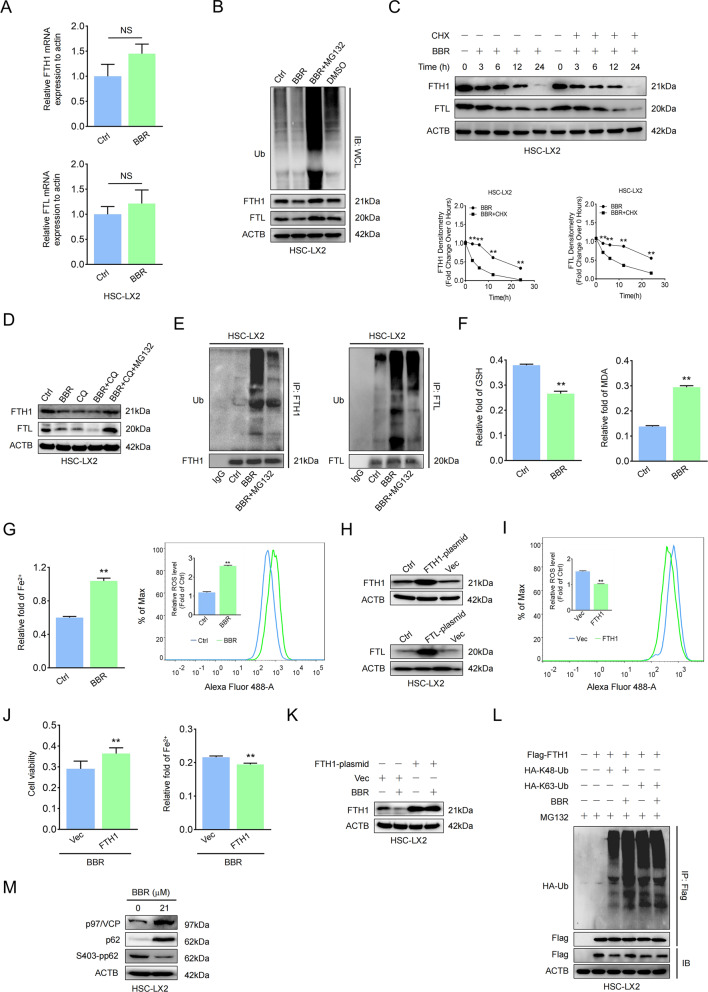
BBR promotes ferritin proteolysis to increase ferrous overload in HSCs. **A** HSC-LX2 cells were treated with BBR (21 μM) for 24 h, and FTH1 and FTL mRNA levels were determined by RT–qPCR. **B** HSC-LX2 cells were exposed to BBR (21 μM) with or without MG-132 (10 μM), and the protein levels of FTH1, FTL, and ubiquitin were determined. **C** HSC-LX2 cells were exposed to BBR (21 μM) with or without cycloheximide (CHX, 100 μM) at the indicated time points, and FTH1 and FTL were assayed. **D** HSC-LX2 cells were treated with BBR (21 μM), CQ (10 μM), or BBR combined with CQ with or without MG-132 (10 μM); FTH1 and FTL levels were determined. **E** HSC-LX2 was treated with BBR (21 μM) in the presence of MG-132 (10 μM); ubiquitinated FTH1 and FTL were precipitated and detected with anti-ubiquitin. **F**, **G** HSC-LX2 cells were exposed to BBR (21 μM) for 24 h; lipid ROS, Fe^2+^, GSH, and MDA were assayed. **H** HSC-LX2 cells were transfected with vector, FTH1, or FTL plasmid; the protein expression of FTH1 and FTL was determined. **I**–**K** HSC-LX2 cells were transfected with vector or FTH1 plasmid and then treated with BBR (21 μM), and ROS, cell viability and Fe^2+^ were investigated. **L** HSC-LX2 cells were transfected with Flag-FTH1, HA- K48-Ub, and HA-K63-Ub plasmids and then treated with BBR (21 μM) and MG-132 (10 μM) for 9 h; an IP assay was used to detect the K48 and K63 sites of FTH1 ubiquitination. **M** HSC-LX2 cells were treated with or without BBR (21 μM); p97/VCP, p62, and S403-pp62 were precipitated. The results are expressed as mean ± SD. Data are representative of three independent experiments; *n* = 3–6 in every group; compared with the control group, **P < 0.01. NS, not significant.

The ALP and UPS are two major cellular degradation machineries, and impaired function of one system influences the other [33, 34]. Whether UPS is a compensatory mechanism for BBR-mediated autophagy dysfunction to act as the housekeeper in modulating HSC ferritin remains unknown. As the data showed, FTH1 and FTL mRNA but not their proteins remained unchanged, indicating no apparent regulation at the transcription level (Fig. 7A and Supplementary Fig. 8A). The addition of MG132, a selective 26S proteasomal degradation inhibitor, blocked the degradation of FTH1 and FTL (Fig. 7B and Supplementary Fig. 8B); however, cycloheximide (CHX), a protein synthesis inhibitor, obviously downregulated FTH1 and FTL, and BBR combined with CHX accelerated the degradation rate of FTH1 and FTL (Fig. 7C and Supplementary Fig. 8C), implying that BBR-modulated ferritin degradation was not acting at the translational level. BBR- or CQ-mediated degradation of ferritin could be reversed by MG132, and the ubiquitylated FTH1 and FTL protein levels were increased after BBR treatment (Fig.7D–G and Supplementary Fig. 8D–G), suggesting that ubiquitin-mediated ferritin degradation could be a potential posttranscriptional mechanism of BBR in regulating ferroptosis in HSCs. Overexpression of the FTH1 plasmid, but not FTL (data not shown), could offset the effect of BBR (Fig. 7H–K). Furthermore, BBR promoted K48- but not K63-linked ubiquitination and degradation of FTH1 through the UPS (Fig. 7L) with enhanced p97/VCP expression and decreased S403-phosphorylated p62 expression (Fig. 7M). Taken together, BBR induces ubiquitin-mediated degradation of ferritin to accelerate iron release and promote HSC ferroptosis.

### Blockage of ferroptosis offsets the effect of BBR against liver fibrosis

**Fig. 8:**
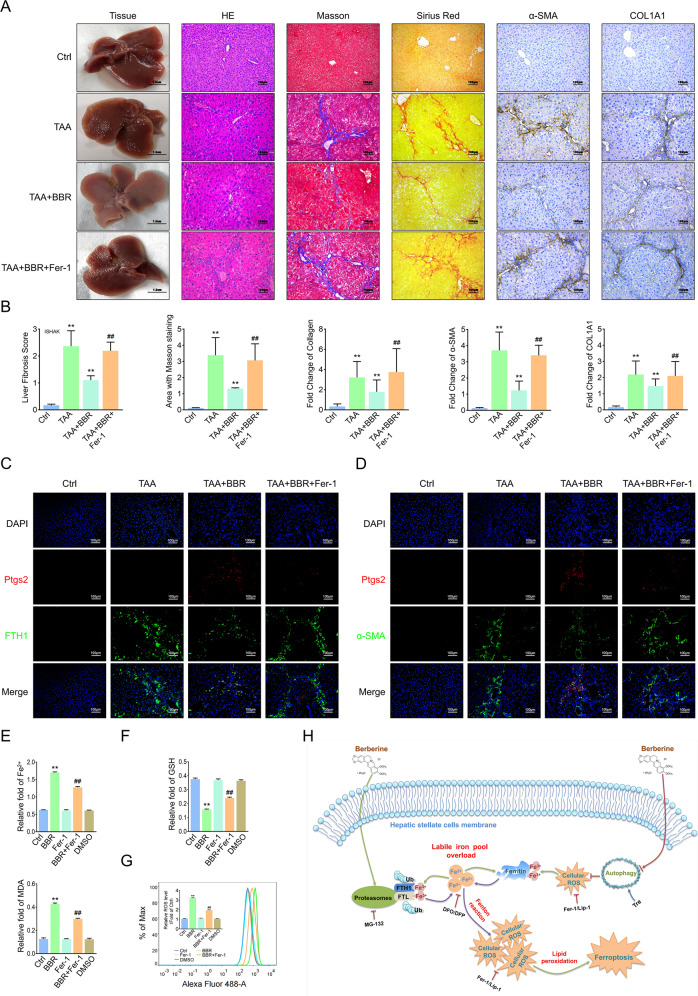
Blockage of ferroptosis offsets the effect of BBR against liver fibrosis. Mice were divided into 4 groups: control group, TAA-treated group, TAA-treated group plus BBR (200 mg/kg/day) with or without Fer-1 (1 mg/kg/day). **A**, **B** General examination of liver tissues by H&E, Masson’s trichrome, and Sirius red staining were performed. Fibrotic biomarkers COL1A1 and α-SMA staining in liver sections (compared with the control group, **P < 0.01; compared with the TAA plus BBR group, ^##^*P* < 0.01). **C**, **D** Mouse liver sections were costained with Ptgs2 and FTH1 or α-SMA. **E**–**G** HSC-LX2 was pretreated with Fer-1 (1 μM) for 1 h; then the cells were treated with BBR (21 μM) for 24 h, and ferroptotic events were investigated. **H** The underlying mechanism of BBR-induced HSC ferroptosis in liver fibrosis. The results are expressed as mean ± SD. Data are representative of three independent experiments; *n* = 3–6 in every group; Scales: 100 μm. Representative photographs are shown. Compared with the control group, **P < 0.01; compared with the BBR group, ^##^*P* < 0.01.

To determine the role of ferroptosis in hepatic fibrosis, we applied Fer-1 to thioacetamide (TAA)-induced mouse liver fibrosis model. BBR obviously alleviated the fibrotic morphology changes pathologically, whereas the antifibrosis efficacy of BBR was almost abolished by Fer-1 (Fig. 8A, B). Costained Ptgs2 with FTH1 or α-SMA in liver sections revealed that BBR treatment markedly downregulated the expression of both FTH1 and α-SMA but significantly enhanced Ptgs2 expression in the collagen deposition area, whereas Fer-1 almost completely abolished the inhibitory effect of BBR on Ptgs2 expression but reversed the BBR-mediated downregulation of FTH1 and α-SMA (Fig. 8C, D). In vitro, decreased HSC activity (Fig. 5A and Supplementary Fig. 6E) and ferroptotic events (Fig. 8E–G and Supplementary Fig. 9A, B) were reversed by Fer-1 treatment. In addition, Fer-1 mediated ferroptosis and fibrosis marker turnover (Fig. 6N and Supplementary Fig. 7N). Taken together, BBR induces HSC ferroptosis, and blockade of ferroptosis counteracts the pharmacological effects of BBR on the liver fibrosis.

## Discussion

Liver fibrosis is a dynamic process of chronic liver inflammation characterized by net accumulation of ECM [1]. If uncontrolled, it would probably progress to liver cirrhosis, liver failure, and even hepatocellular carcinoma [2]. Activation of HSCs into myofibroblasts has been established as the main driver of fibrosis. However, the lack of effective treatments to reverse the process remains a major challenge for liver fibrosis patients. Here we found that BBR treatment rescued liver fibrosis by inducing HSC ferroptosis. Mechanistically, BBR-induced autophagy inhibition in HSCs resulted in elevated ROS and increased oxidative stress, triggering the degradation of ferritin and enhancing redox-active iron accumulation; the latter increased the risk of amplifying further ROS generation through the Fenton reaction. Moreover, impaired autophagy compensation enhanced the function of the UPS and led to ferritin proteolysis, further aggravated iron homeostasis disruption, and promoted HSC ferroptosis.

Ferroptosis, a newly discovered novel type of RCD, is implicated in metabolism and redox signaling as well as diverse pathophysiological conditions [35, 36], including liver fibrosis. However, the function of ferroptosis in liver fibrosis remains controversial. Some studies have shown that ferroptosis plays an antifibrotic role in hepatic fibrosis. Li et al. showed artemether-induced HSC ferroptosis through the IRP2–iron–ROS axis by correcting iron metabolism [37]. Zhang et al. found that ferroptosis was involved in the process of relieving liver fibrosis through activating autophagy by RNA-binding protein [26]. However, others hold the opposite opinion. Yu et al. fed mice a high-iron diet to increase their susceptibility to liver fibrosis by inducing ferroptosis, which was reversed by ferroptosis inhibitor, indicating that ferroptosis contributed to liver fibrosis [25]. However, ferroptosis in liver fibrosis does not always cause the remission of fibrotics, perhaps its function largely depends on cell types or target protein load.

The current study indicated that BBR significantly decreased HSC viability and resulted in cell death, whereas ferroptosis inhibitors or iron chelators, but not apoptosis or necroptosis inhibitors, diminished the promoting effect of BBR on cell death. Although different concentrations of BBR may elicit different types of cell death at different time points, it is therefore possible that ferroptosis might be intertwined with other cell death pathways in the pathophysiological process of relieving liver fibrosis, including but not limited to other forms of RCDs, for example, as a part of pathogenic amplification loops. However, here we clearly demonstrated that a certain proportion of BBR-induced HSC death was attributable to ferroptosis and was predominantly responsible for BBR-induced HSC death. Moreover, we confirmed that the indicated concentration of BBR neither impaired hepatocyte cell viability and cell proliferation nor induced mouse liver injury; that is, a certain concentration of BBR specifically induced HSC ferroptosis to alleviate liver fibrosis. BBR-induced ferroptosis in activated HSCs provides a brake on the fibrogenic response to damage by suppressing the activation and proliferation of HSCs to produce fibrous scars.

Autophagy is a self-degradation intracellular process involving intracellular substrate degradation and acts as a housekeeper in the maintenance of cellular homeostasis [38]. Autophagy-induced HSC activation results in the release of ECM and promotes the progression of fibrosis [3, 5, 30], whereas blocking autophagy leads to HSC activation suppression and fibrosis attenuation. ROS is critical for many cellular processes, and the generation and scavenging of ROS needs to be precisely regulated to maintain the balance of redox [11]. Autophagy is one of the most important mechanisms of ROS modulation, and a homeostatic link between autophagy and ROS is important for physiological and pathological conditions in cellular responses. Cells deficient in the essential autophagy gene Atg7 have increased basal levels of ROS [15], and subsequently, this induces cell death, including ferroptosis [39].

Iron metabolism is important in the liver [40]; as an essential mineral, it is involved in various biological processes; and either deficiency or excess is detrimental [41]. In normal physiological conditions, iron is present within cells as low-molecular-weight iron chelates, heme-associated iron, or ferritin-bound iron. The majority of excess intracellular iron is rapidly sequestered and safely stored in a ferric state (Fe^3+^) in ferritin [22], a 24-subunit polymer comprised of two similar polypeptides, FTH1 and FTL, the ratios of which vary among different tissue types: FTL-rich ferritin is expressed in large quantities in iron-storage organs such as the liver and spleen, whereas FTH1-rich ferritin is abundant in organs with low iron content, such as the heart [22, 42]. Increased ferritin expression enhances cell growth and improves resistance to oxidative stress [43] and reduces ferroptosis [44]. Recent studies indicate that increased autophagy can degrade ferritin through ATG5-ATG7-NCOA4-mediated ferritinophagy and enhance intracellular ferrous iron levels, resulting in oxidative injury by Fenton reaction [23, 32]. However, superoxide anion radicals have been confirmed as another important reductive inducer to mobilize the iron from ferritin [21, 45–48]. Numerous studies have confirmed that ferritin-bound ferric iron (Fe^3+^) can be reduced to its ferrous state (Fe^2+^) by the action of certain reductants, including superoxide, and be released from ferritin during ferritin degradation triggered by superoxide [20, 21, 49]; once released, these liberated Fe^2+^ ions are transported to the cytosol to form the cytosolic part of redox-active iron to take part in the redox cycle to produce Fenton reaction and promote peroxidation of phospholipid liposomes, which in turn accelerate the disintegration of ferritin [21] and promote cell ferroptosis.

Accumulating iron content plays an important role in ferroptosis [24]. Chen et al. revealed that artemisinin compounds sensitized cancer cells to ferroptosis by regulating cellular iron homeostasis [28]. Brown et al. demonstrated that prominin2 drives ferroptosis resistance in cells by stimulating iron export [50]. Chen et al. suggested the ATM-MTF1-ferritin/FPN1 regulatory axis was a novel determinant that modulates ferroptosis by regulating labile iron levels [51]. Liu et al. found that NUPR1-mediated LCN2 expression blocked ferroptotic cell death by diminishing iron accumulation [52].

In our study, we demonstrated that BBR-induced HSC ferroptosis was involved in the process of modulating iron homeostasis by disintegrating ferritin; however, this could be reversed by autophagy inducers, lipid ROS scavengers or iron chelators, indicating that BBR downregulated autophagy to enhance ROS/Fe^2+^-mediated HSC ferroptosis. Mechanistically, BBR-induced autophagy suppression was incapable of eliminating the elevated ROS, which increased oxidative stress in HSCs, creating a vicious feed-forward loop, which resulted in ferritin degradation and triggered HSC ferroptosis. These data are consistent with previous findings [20, 21, 49]. However, whether the released iron was derived from FTH1 or FTL is still unclear.

The ALP and UPS are two major cellular degradation machineries in eukaryotes [33, 34]. Ubiquitylation has been proposed to be a common constituent that directs substrates to proper degradation systems and even precipitates UPS-autophagy crosstalk [53]. Suppression of autophagy by knockout of essential autophagic genes Atg5 or Atg7 in mice resulted in the accumulation of ubiquitylated proteins [34, 54]; however, in our study, autophagy-defective HSCs did not aggregate ubiquitylated ferritin and instead degraded it in a proteasome-dependent manner. We found that BBR treatment promoted K48- but not K63-linked ubiquitination and degradation of FTH1 through the UPS. Mechanistically, BBR downregulated 403-phosphorylated p62, which might lead to a decrease in ubiquitylated protein targeting to sequestosomes for autophagic degradation. In addition, BBR enhanced p97/VCP expression, and the latter led to sequestration of ubiquitylated ferritin, which were otherwise p62 targets, consistent with previous reports [53–55]. Taken together, BBR suppressed HSC autophagy, directed ubiquitylated ferritin for degradation by the UPS, and promoted HSC ferroptosis.

ALP and UPS add complexity to the control of iron homeostasis by BBR, and our results raise a series of questions: do ALP and UPS play a major role in the process of iron regulation in the pathophysiological processes of BBR-treated liver fibrosis? Were there other independent forms involved in the modulation of ferritin besides the existing ones, for example, by pupylation of ferritin, like in *Corynebacterium glutamicum* [56]? The exact mechanisms remain to be further investigated and characterized.

In conclusion, our findings confirmed that BBR induced iron disruption in HSCs by modulating ferritin degradation in both the autophagy/ROS and UPS pathways, driving HSC ferroptosis to attenuate liver fibrosis (Fig. 8H). The new discovered link among BBR, ferritin, and iron opens up a sense of perspective about the potential pharmacological role of BBR in the mechanism of iron release from ferritin and sheds light on the treatment of liver fibrosis.

## Materials and methods

### Human liver tissue samples

Nine normal liver tissue samples were collected from parahemangioma sites of hepatic hemangioma patients without hepatitis, and paired fibrosis samples were obtained from hepatitis B virus-infected, alcohol-induced, and secondary biliary liver fibrosis patients during operations before any therapeutic intervention. All of these tissues were diagnosed by histopathology. The study protocol was approved by the Clinical Research Ethics Committee of The Third Affiliated Hospital of Sun Yat-Sen University. Written informed consent was obtained from each study participant before this study.

### Study approval and mouse treatment

All animal experiments were conducted at The Third Affiliated Hospital of Sun Yat-Sen University in accordance with the protocol approved by the Institutional Animal Care and Use Committee of Sun Yat-Sen University (IACUC-F3-19-0311). Animal studies are reported in compliance with the ARRIVE guidelines [57]. A total of 120 C57BL/6 mice were used for animal experiments in a blinded manner. Briefly, 6-week-old male mice (20–25 g) were randomly allocated to the indicated groups and housed in cages with ad libitum access to food and water, following habituation to a 12 h light/dark cycle. The experimenter was blinded to the group allocation. The mouse liver fibrosis model group was established via intraperitoneal (i.p.) injection with TAA or carbon tetrachloride (CCl_4_) for 6 weeks [58, 59]. The control group was injected i.p. with the same volume of olive oil or saline only. For the BBR group, a dose of BBR (200 mg/kg/day) was given by oral gavage [10]. In addition to the i.p. injection. with TAA or CCl_4_, the mice in the treatment group received BBR intragastrically. In the inhibitor group, besides the injected i.p. with TAA and BBR oral gavage treatment, mice were injected i.p. with a dose of Fer-1 (1 mg/kg/day). Mice were sacrificed under 3% isoflurane anesthesia after 6 weeks of treatment; blood and liver tissue samples were taken and processed for subsequent analyses. At least five mouse hepatic sections were utilized in every group.

### Antibodies

Antibodies against COL1A1 (ab34710), COL4A1 (ab6586), α-SMA (ab5694/ab7817), vimentin (ab92547), p75^NTR^ (ab52987), desmin (ab32362), xCT (ab175186), Ptgs2 (ab179800), GPx4 (ab125066), FTH1 (ab65080/ab183781), FTL (ab69090), Atg5 (ab108327), Atg7 (ab133528), LC3B (ab48394), and 4-HNE (ab46544) were purchased from Abcam Technology (Abcam, Cambridge, UK). Antibodies against FTH1 (sc-376594), FTL (sc-390558), and ubiquitin (sc-8017 AC) were purchased from Santa Cruz Biotechnology (Santa Cruz, CA, USA). Antibodies against α-SMA (14395-1-AP), Ptgs2 (66351-1-Ig), BECN1 (11306-1-AP), p97/VCP (10736-1-AP), and SQSTM1/p62 (18420-1-AP) were purchased from Proteintech (Proteintech, IL, USA). Horseradish peroxidase (HRP)-conjugated AffiniPure goat anti-mouse IgG light chain (AS062), HRP-conjugated goat anti-mouse IgG heavy chain (AS064), and anti-NCOA4 (A5695) were purchased from ABclonal (ABclonal, Wuhan, China). Antibodies against S403-pp62 (#39786), LC3B (#83506), PCNA (#13110), and GAPDH (#5174) were purchased from Cell Signaling Technology (Cell Signaling Technology, Danvers, MA, USA). Monoclonal Anti-FLAG® M2 (F1804), Monoclonal Anti-HA (H9658), and anti-ACTB (A5441) were purchased from Sigma–Aldrich (Sigma–Aldrich, St. Louis, MO, USA).

### Chemicals

Berberine (PHR1502), trehalose (T0167), CCl_4_ (SHBF2363V), TAA (BCBW8069), and potassium hexacyanoferrate (II) trihydrate (P9387) were purchased from Sigma–Aldrich (St. Louis, MO, USA). CQ (S4157), 3-MA (S2767), deferoxamine (S5742), deferiprone (S4067), liproxstatin-1 (S7699), necrostatin-1 (S8037), Z-VAD-FMK (S7023), calpeptin (S7396), RSL3 (S8155), sorafenib (S7397), and erastin (S7242) were obtained from Selleck Chemicals (Selleck, Houston, TX, USA). Fluorescein diacetate (HY-D0719), ferrostatin-1 (HY-100579), SBE-β-CD (HY-17031), and CHX (HY-12320) were purchased from MCE (MCE, Washington, USA). MG-132 (tlrl-mg132) was purchased from Invitrogen (Invitrogen, CA, USA). Ammonium ferric citrate was purchased from Macklin (C10792850, Macklin, Shanghai, China).

### Cell culture and transfection

The human HSC line (HSC-LX2), hepatocyte cell line (LO2), and rat HSC line (HSC-T6) were obtained from American Type Culture Collection (ATCC, USA). The rat hepatocyte cell line (BRL-3A) and GFP-LC3B were kindly provided by Dr. Dongbo Qiu (Department of The Biological Therapy Center, The Third Affiliated Hospital of Sun Yat-Sen University, Guangzhou, China). All cells were cultured in Dulbecco’s modified Eagle’s medium (Thermo Scientific, Rockford, AL, USA) supplemented with 10% fetal bovine serum (Gibco-BRL, Grand Island, NY, USA), 2 mM L-glutamine, and 1% penicillin–streptomycin (Gibco-BRL). The cells were dissociated with 0.05% trypsin and counted with a TC20™ automated cell counter (Thermo Scientific). Plasmids encoding the pcDNA3.0-HA-K48/K63-Ub-vector were kindly provided by Professor Yunfei Qin (Department of The Biological Therapy Center, The Third Affiliated Hospital of Sun Yat-Sen University, Guangzhou, China). FTH1, FTL, FTH1-Flag, and vector plasmids were created and synthesized by Genecopoeia (Guangzhou, China). Before transfection, HSC-LX2 cells were transplanted into 6-well or 96-well plates and grown to a confluence of 50–60%. Lipofectamine^TM^ 3000 (Thermo Fisher Scientific, Waltham, MA, USA) was used according to the manufacturer’s instructions. The pcDNA3.0-Vector was transfected as the negative control. All plasmids were incubated with the transfection media for 24 h before the following experiments.

### Total RNA extraction and real-time PCR

Total RNA was isolated from HSCs using TRIzol reagent (Invitrogen, Carlsbad, CA, USA) according to the manufacturer’s protocol. Then 2 µg of total RNA was used for cDNA synthesis using the iScript™ cDNA Synthesis Kit (Bio-Rad, California, USA). qPCR was carried out using iQSYBR Green Master Mix with a Bio-Rad CFX96 Real-Time System. The gene expression levels were normalized to β-actin. Relative expression levels were calculated by the 2^−ΔΔCt^ method. The primer sequences used for the qPCR are listed in Supplementary Table 1.

### Western blotting

Protein was extracted from cells or mouse liver tissues with lysis buffer (P0013, Beyotime Institute of Biotechnology, Jiangsu, China) supplemented with protease inhibitor cocktail (04693116001; Roche Diagnostics, Indianapolis, IN, USA) and phosphatase inhibitor cocktail (78420; Thermo Scientific, Waltham, MA), and the samples were then centrifuged. Immunoblotting was performed as described previously [3]. Immunodetection was achieved with Hyglo chemiluminescence reagent (Denville Scientific) and detected by a Bio-Rad ECL machine (Bio-Rad). After signal quantification by densitometry, the results were expressed as a ratio to the loading control in densitometry units.

### Immunoprecipitation (IP)

IP was performed using the Thermo Scientific Pierce IP Kit (Thermo Scientific). Briefly, after treatments, cultured cells were washed in cold phosphate-buffered saline (PBS) three times and then lysed with lysis buffer. IP antibody (5 μg) or normal IgG was added and incubated at 4 °C overnight on a rotator. In all, 25 µl of A/G agarose beads were added to 200 µl lysates and incubated for 3 h with gentle agitation at room temperature, according to the manufacturer’s protocol. The beads were removed by centrifugation, and the final samples were analyzed by western blot using the indicated antibodies.

### Liver histologic and HSC staining

Masson’s trichrome staining, Sirius red, H&E, immunohistochemical (IHC), and IF staining, and double IF staining were performed as previously described [3]. Masson’s trichrome staining and Sirius red staining were used for collagen investigation, and semiquantitative analysis was performed using Image-Pro Plus 8.0 (Media Cybernetics, Rockville, MD, USA). For IHC, after antigen retrieval, 3 μm-thick paraffin-embedded sections were permeabilized with 1% Triton X-100, followed by PBS washes and incubation with the indicated antibodies overnight at 4 °C. Next, the targeted protein was detected using a secondary antibody and then incubated in DAB for intensification, and the sections were counterstained with hematoxylin. For IF staining, the targeted proteins were incubated with the indicated primary antibodies overnight at 4 °C, followed by the related biotin-conjugated secondary antibody and streptavidin Alexa Fluor 488® or 594® (Thermo Fisher Scientific). The nuclei were stained with 4,6-diamidino-2-phenylindole (DAPI). For double staining, after completing the first protein staining, the sections were then re-incubated, and the secondary protein was detected. The liver fibrosis stage was assessed by the Ishak scale [60].

### Perls’-DAB staining

Liver tissue sections were histochemically incubated in Perls’ solution (5% potassium ferrocyanide/5% hydrochloric acid) for 30 min. Then the sections were washed with PBS 3 times, incubated in DAB for intensification, and washed with deionized water to stop the reaction. Next, the sections were counterstained with hematoxylin.

### Examination of GFP-LC3B and p62

HSC-LX2 cells were cultured on confocal plates and transiently transfected with GFP-LC3B according to the manufacturer’s instructions. The cells were incubated with the indicated chemicals, and then Hoechst 33342 (Thermo Fisher Scientific) reaction solution was added to detect the staining. For p62 detection, HSC-LX2 cells expressing GFP-LC3B were treated with the indicated chemicals and then fixed with 4% formaldehyde for 30 min. Anti-p62 antibody (1:200) was added to the cells and incubated overnight at 4 °C. Cells were washed with PBS, Alexa Fluor 594® (Thermo Fisher Scientific) was added and incubated for 2 h, and the nuclei were stained with DAPI. LC3B puncta and p62 were observed under a Zeiss LSM 880 confocal microscope (Zeiss, Oberkochen, Germany).

### Transmission electron microscopy

HSCs were pretreated with or without BBR and then fixed with ice-cold 2.5% glutaral. TEM was performed using a transmission electron microscope (JEOL, Tokyo, Japan) at the electron microscopy core laboratory of Sun Yat-Sen University.

### Liver function assay

For the liver function test, the serum concentrations of transaminase aspartate aminotransferase and alanine aminotransferase in the indicated samples were detected with enzyme-linked immunosorbent assay (ELISA) kits (Cusabio, Wuhan Huamei Biotech, Wuhan, China) according to the manufacturer’s instructions. Lactic dehydrogenase, hyaluronic acid, procollagen III, and collagen IV in the serum of the mice were determined by ELISA kits (Sinopharm Chemical Reagent).

### Cell death measurement

HSC necrotic cell death and live cells were indicated by PI staining (KeyGEN BioTECH, Nanjing, China) and FDA staining (MCE) according to the manufacturer’s instructions.

### Cell viability assay and 5-ethynyl-2′-deoxyuridine (EdU) assay

Cell viability was detected by a Cell Counting Kit-8 (CCK-8) assay (Dojindo Laboratories, JAPAN) according to the manufacturer’s instructions. Briefly, HSCs and hepatocytes were seeded into 96-well plates at a density of 2500 cells per well. CCK-8 reagent (10 μl/100 μl medium) was added to each well after the cells were treated with the indicated chemicals. After incubation for 2 h, the optical density (OD) was measured at 550 nm. The cell proliferation rate was detected by an EdU assay (RiboBio Co., Ltd., Guangzhou, China) according to the manufacturer’s instructions. Briefly, cells were seeded into 48-well plates and incubated with the IC50 concentration of BBR for the indicated time. Each well was incubated with 50 μM EdU solution for 2 h, fixed with 4% paraformaldehyde for 30 min, incubated with 2 mg/ml glycine solution for 5 min to decolor on a shaker, and then washed with cold PBS containing 0.5% Triton X-100 for 10 min. Nuclear staining was performed with the addition of Apollo® staining solution followed by incubation in the dark and microscopic inspection (Zeiss).

### Malondialdehyde (MDA) assay

The relative MDA concentration in the cell lysates was measured using a lipid peroxidation assay kit (Abcam, ab118970) according to the manufacturer’s instructions. Briefly, MDA in HSCs reacted with thiobarbituric acid (TBA) to generate the MDA-TBA adduct, and the OD of each pore was measured at 532 nm by a spectrophotometer (BioTek-Epoch2, USA).

### Iron assay

The relative cellular ferrous iron (Fe^2+^) level in the cell lysates was checked with an iron assay kit (Abcam, ab83366) according to the manufacturer’s instructions. Briefly, after treatments, HSCs were collected and washed in cold PBS three times. Then, 200 µl iron assay buffer was added to each well of the cells on ice. Cell lysates were collected, and iron reducer was added to reduce the switch from Fe^3+^ to Fe^2+^, mixed carefully, and reacted for 30 min. Subsequently, the iron probe was added and mixed thoroughly and then incubated for 60 min. The output was measured immediately at 593 nm by a spectrophotometer (BioTek-Epoch2).

### Detection of lipid ROS

Lipid ROS level was determined using 5 µM C11-BODIPY 488 dye [35]. Briefly, cells were seeded in 6-well plates, treated with or without the indicated concentrations of BBR for 24 h, with or without either the indicated inhibitors or agonists. Twenty-four hours later, the culture medium was replaced with 2 ml of C11-BODIPY 488 medium. After incubation for another 15 min, the cells were harvested in 15 ml tubes and washed with PBS twice. Then the cells were resuspended in 500 µl PBS. The cell suspension was filtered through a cell strainer (40 µm nylon mesh) and subjected to flow cytometric analysis to examine the amount of ROS within the cells. The fluorescence intensities of the cells per sample were determined by flow cytometry using a BD FACSAria flow cytometer (BD Biosciences).

### Glutathione (GSH) assay

The GSH concentration in the cell lysates was determined using a GSH assay kit (Sigma, CS0260) according to the manufacturer’s instructions. Briefly, after treatments, HSCs were homogenized in 50 mM MES buffer (Sigma–Aldrich, M8250) containing 1 mM EDTA (Sigma–Aldrich, 03620). The samples were centrifuged at 10,000 × *g* for 10 min at 4 °C, the supernatants were subjected to the GSH assay kit and mixed with GSH detection working solution along with standards, and the samples were incubated for 25 min at room temperature. Then the output was measured immediately at 412 nm by a spectrophotometer (BioTek-Epoch2).

### Statistical analysis

All experiments were independently repeated at least three times. Data are represented as mean ± SD. The statistical significance was evaluated by using one-way analysis of variance tests or Student’s *t* tests, and all tests were two-tailed. All data were analyzed by GraphPad Prism 8.0 (GraphPad Software, Inc.). *P* < 0.05 was considered statistically significant.

## Supplementary information

cddiscovery-author-contribution

Supplementary table 1

Supplementary Figure legends

Supplementary Fig. 1

Supplementary Fig. 2

Supplementary Fig. 3

Supplementary Fig. 4

Supplementary Fig. 5

Supplementary Fig. 6

Supplementary Fig. 7

Supplementary Fig. 8

Supplementary Fig. 9

## Data Availability

The datasets used and/or analyzed during the current study are available from the corresponding author on reasonable request.

## References

[1] Kisseleva T, Brenner D (2021). Molecular and cellular mechanisms of liver fibrosis and its regression. Nat Rev Gastroenterol Hepatol.

[2] Garrido A, Djouder N (2021). Cirrhosis: a questioned risk factor for hepatocellular carcinoma. Trends Cancer.

[3] Tan S, Lu Y, Xu M, Huang X, Liu H, Jiang J (2019). beta-Arrestin1 enhances liver fibrosis through autophagy-mediated snail signaling. FASEB J.

[4] Marti-Rodrigo A, Alegre F, Moragrega AB, Garcia-Garcia F, Marti-Rodrigo P, Fernandez-Iglesias A (2020). Rilpivirine attenuates liver fibrosis through selective STAT1-mediated apoptosis in hepatic stellate cells. Gut..

[5] Thoen LF, Guimaraes EL, Dolle L, Mannaerts I, Najimi M, Sokal E (2011). A role for autophagy during hepatic stellate cell activation. J Hepatol.

[6] Barcena-Varela M, Paish H, Alvarez L, Uriarte I, Latasa MU, Santamaria E (2021). Epigenetic mechanisms and metabolic reprogramming in fibrogenesis: dual targeting of G9a and DNMT1 for the inhibition of liver fibrosis. Gut..

[7] Habtemariam S (2016). Berberine and inflammatory bowel disease: a concise review. Pharm Res.

[8] Yin J, Xing H, Ye J (2008). Efficacy of berberine in patients with type 2 diabetes mellitus. Metabolism..

[9] Meeran SM, Katiyar S, Katiyar SK (2008). Berberine-induced apoptosis in human prostate cancer cells is initiated by reactive oxygen species generation. Toxicol Appl Pharmacol.

[10] Sun X, Zhang X, Hu H, Lu Y, Chen J, Yasuda K (2009). Berberine inhibits hepatic stellate cell proliferation and prevents experimental liver fibrosis. Biol Pharm Bull.

[11] Moloney JN, Cotter TG (2018). ROS signalling in the biology of cancer. Semin Cell Dev Biol.

[12] Scherz-Shouval R, Shvets E, Fass E, Shorer H, Gil L, Elazar Z (2007). Reactive oxygen species are essential for autophagy and specifically regulate the activity of Atg4. EMBO J.

[13] Lee IH, Kawai Y, Fergusson MM, Rovira II, Bishop AJ, Motoyama N (2012). Atg7 modulates p53 activity to regulate cell cycle and survival during metabolic stress. Science.

[14] Kroemer G, Marino G, Levine B (2010). Autophagy and the integrated stress response. Mol Cell.

[15] Filomeni G, De Zio D, Cecconi F (2015). Oxidative stress and autophagy: the clash between damage and metabolic needs. Cell Death Differ.

[16] Mathew R, Karp CM, Beaudoin B, Vuong N, Chen G, Chen HY (2009). Autophagy suppresses tumorigenesis through elimination of p62. Cell..

[17] Stockwell BR, Friedmann Angeli JP, Bayir H, Bush AI, Conrad M, Dixon SJ (2017). Ferroptosis: a regulated cell death nexus linking metabolism, redox biology, and disease. Cell..

[18] Bogdan AR, Miyazawa M, Hashimoto K, Tsuji Y (2016). Regulators of iron homeostasis: new players in metabolism, cell death, and disease. Trends Biochem Sci.

[19] Verbon EH, Trapet PL, Stringlis IA, Kruijs S, Bakker P, Pieterse CMJ (2017). Iron and immunity. Annu Rev Phytopathol.

[20] Dognin J, Crichton RR (1975). Mobilisation of iron from ferritin fractions of defined iron content by biological reductants. FEBS Lett.

[21] Thomas CE, Morehouse LA, Aust SD (1985). Ferritin and superoxide-dependent lipid peroxidation. J Biol Chem.

[22] Linder MC (2013). Mobilization of stored iron in mammals: a review. Nutrients..

[23] Hou W, Xie Y, Song X, Sun X, Lotze MT, Zeh HJ (2016). Autophagy promotes ferroptosis by degradation of ferritin. Autophagy..

[24] Dixon SJ, Lemberg KM, Lamprecht MR, Skouta R, Zaitsev EM, Gleason CE (2012). Ferroptosis: an iron-dependent form of nonapoptotic cell death. Cell..

[25] Yu Y, Jiang L, Wang H, Shen Z, Cheng Q, Zhang P (2020). Hepatic transferrin plays a role in systemic iron homeostasis and liver ferroptosis. Blood..

[26] Zhang Z, Guo M, Li Y, Shen M, Kong D, Shao J (2020). RNA-binding protein ZFP36/TTP protects against ferroptosis by regulating autophagy signaling pathway in hepatic stellate cells. Autophagy..

[27] Passino MA, Adams RA, Sikorski SL, Akassoglou K (2007). Regulation of hepatic stellate cell differentiation by the neurotrophin receptor p75NTR. Science..

[28] Chen G, Benthani F, Wu J, Liang D, Bian Z, Jiang XJ (2020). Artemisinin compounds sensitize cancer cells to ferroptosis by regulating iron homeostasis. Cell Death Differ.

[29] Bertrand RL (2017). Iron accumulation, glutathione depletion, and lipid peroxidation must occur simultaneously during ferroptosis and are mutually amplifying events. Med Hypotheses.

[30] Hernandez-Gea V, Ghiassi-Nejad Z, Rozenfeld R, Gordon R, Fiel MI, Yue Z (2012). Autophagy releases lipid that promotes fibrogenesis by activated hepatic stellate cells in mice and in human tissues. Gastroenterology.

[31] Li YC, Zhang MQ, Zhang JP (2018). Opposite effects of two human ATG10 isoforms on replication of a HCV sub-genomic replicon are mediated via regulating autophagy flux in zebrafish. Front Cell Infect Microbiol.

[32] Mancias JD, Wang X, Gygi SP, Harper JW, Kimmelman AC (2014). Quantitative proteomics identifies NCOA4 as the cargo receptor mediating ferritinophagy. Nature..

[33] Kocaturk NM, Gozuacik D (2018). Crosstalk between mammalian autophagy and the ubiquitin-proteasome system. Front Cell Dev Biol.

[34] Hara T, Nakamura K, Matsui M, Yamamoto A, Nakahara Y, Suzuki-Migishima R (2006). Suppression of basal autophagy in neural cells causes neurodegenerative disease in mice. Nature..

[35] Xu M, Tao J, Yang Y, Tan S, Liu H, Jiang J (2020). Ferroptosis involves in intestinal epithelial cell death in ulcerative colitis. Cell Death Dis.

[36] Fang X, Cai Z, Wang H, Han D, Cheng Q, Zhang P (2020). Loss of cardiac ferritin H facilitates cardiomyopathy via Slc7a11-mediated ferroptosis. Circ Res.

[37] Li Y, Jin C, Shen M, Wang Z, Tan S, Chen A (2020). Iron regulatory protein 2 is required for artemether -mediated anti-hepatic fibrosis through ferroptosis pathway. Free Radic Biol Med.

[38] Zhang XW, Zhou JC, Peng D, Hua F, Li K, Yu JJ (2020). Disrupting the TRIB3-SQSTM1 interaction reduces liver fibrosis by restoring autophagy and suppressing exosome-mediated HSC activation. Autophagy..

[39] Su LJ, Zhang JH, Gomez H, Murugan R, Hong X, Xu D (2019). Reactive oxygen species-induced lipid peroxidation in apoptosis, autophagy, and ferroptosis. Oxid Med Cell Longev.

[40] Wang C, Babitt JJB (2019). Liver iron sensing and body iron homeostasis. Blood..

[41] Dixon SJ, Stockwell BR (2014). The role of iron and reactive oxygen species in cell death. Nat Chem Biol.

[42] Ganz T (2013). Systemic iron homeostasis. Physiol Rev.

[43] Baldi A, Lombardi D, Russo P, Palescandolo E, De Luca A, Santini D (2005). Ferritin contributes to melanoma progression by modulating cell growth and sensitivity to oxidative stress. Clin Cancer Res.

[44] Yang WS, Stockwell BR (2008). Synthetic lethal screening identifies compounds activating iron-dependent, nonapoptotic cell death in oncogenic-RAS-harboring cancer cells. Chem Biol.

[45] Cassanelli S, Moulis J (2001). Sulfide is an efficient iron releasing agent for mammalian ferritins. Biochim Biophys Acta.

[46] Double KL, Maywald M, Schmittel M, Riederer P, Gerlach M (1998). In vitro studies of ferritin iron release and neurotoxicity. J Neurochem.

[47] Monteiro HP, Winterbourn CC (1988). The superoxide-dependent transfer of iron from ferritin to transferrin and lactoferrin. Biochem J.

[48] Bolann BJ, Ulvik RJ (1987). Release of iron from ferritin by xanthine oxidase. Role of the superoxide radical. Biochem J.

[49] Yoshida T, Tanaka M, Sotomatsu A, Hirai S (1995). Activated microglia cause superoxide-mediated release of iron from ferritin. Neurosci Lett.

[50] Brown CW, Amante JJ, Chhoy P, Elaimy AL, Liu H, Zhu LJ (2019). Prominin2 drives ferroptosis resistance by stimulating iron export. Dev Cell.

[51] Chen PH, Wu J, Ding CC, Lin CC, Pan S, Bossa N (2020). Kinome screen of ferroptosis reveals a novel role of ATM in regulating iron metabolism. Cell Death Differ.

[52] Liu J, Song X, Kuang F, Zhang Q, Xie Y, Kang R (2021). NUPR1 is a critical repressor of ferroptosis. Nat Commun.

[53] Dikic I (2017). Proteasomal and autophagic degradation systems. Annu Rev Biochem.

[54] Korolchuk VI, Mansilla A, Menzies FM, Rubinsztein DC (2009). Autophagy inhibition compromises degradation of ubiquitin-proteasome pathway substrates. Mol Cell.

[55] Matsumoto G, Wada K, Okuno M, Kurosawa M, Nukina N (2011). Serine 403 phosphorylation of p62/SQSTM1 regulates selective autophagic clearance of ubiquitinated proteins. Mol Cell.

[56] Kuberl A, Polen T, Bott M (2016). The pupylation machinery is involved in iron homeostasis by targeting the iron storage protein ferritin. Proc Natl Acad Sci USA.

[57] Kilkenny C, Browne WJ, Cuthill IC, Emerson M, Altman DG (2010). Improving bioscience research reporting: the ARRIVE guidelines for reporting animal research. PLoS Biol.

[58] Seki E, de Minicis S, Inokuchi S, Taura K, Miyai K, van Rooijen N (2009). CCR2 promotes hepatic fibrosis in mice. Hepatology..

[59] Popov Y, Sverdlov DY, Sharma AK, Bhaskar KR, Li S, Freitag TL (2011). Tissue transglutaminase does not affect fibrotic matrix stability or regression of liver fibrosis in mice. Gastroenterology..

[60] Standish RA, Cholongitas E, Dhillon A, Burroughs AK, Dhillon AP (2006). An appraisal of the histopathological assessment of liver fibrosis. Gut..

