# Supplementary material for: Berberine alleviates liver fibrosis through inducing ferrous redox to activate ROS-mediated hepatic stellate cells ferroptosis
Source: Cell Death Discov. 2021 Dec 4;7:374. doi: 10.1038/s41420-021-00768-7 (PMC8643357; doi:10.1038/s41420-021-00768-7)
Supplement: Supplementary file 1 — cddiscovery-author-contribution [file 41420_2021_768_MOESM1_ESM.pdf]

DECLARATION OF CONTRIBUTIONS TO ARTICLE

ADMC

Manuscript Number:	Journal Name:	
CDDISCOVERY-21-2532R1	*Cell Death Discovery*	(the 'Journal')

Proposed Title of the Contribution:

Berberine alleviates liver fibrosis through inducing ferrous redox to activate ROS-mediated hepatic stellate cells ferroptosis (the 'Contribution)

Author(s):

Jiazhi Yi, Shuyun Wu, Siwei Tan, Yunfei Qin, Xing Wang, Jie Jiang, Huiling Liu, Bin Wu* (the 'Authors')

For all *CDDis*covery articles, each person named as an author in the published version must be able to show he or she has contributed substantially to the article.

Authorship credit should be based on 1) substantial contributions to conception and design, acquisition of data, or analysis and interpretation of data; 2) drafting the article or revising it critically for important intellectual content; and 3) final approval of the version to be published. Authors should meet conditions 1, 2 and 3.

Any person who cannot be shown to have made a substantial contribution to the article cannot be listed as an author in the final version. The name of any person who is deemed to have made a minor contribution can, however, appear in the Acknowledgments section of the article.

Please complete the table below to indicate the contributions of all named authors to the manuscript.

Author Full Name:	Specification of Contribution to the Manuscript:
Jiazhi Yi	Designed the study, performed the experiments, conducted data analysis, made figures, and wrote the manuscript
Shuyun Wu	Performed the experiments, conducted data analysis, made figures and wrote the manuscript.
Siwei Tan	Collected the samples.
Yunfei Qin	Collected the samples.
Xing Wang	Collected the samples.
Jie Jiang	Raised all the mice, and conducted administrative and material support.
Huiling Liu	Raised all the mice, and conducted administrative and material support.
Bin Wu	Designed the study, conducted data analysis, wrote the manuscript, supervised the project and critically revised the manuscript.

Please complete the table below to indicate the contributions of all named authors to the figures.

Figures 1-6:

Jiazhi Yi

Figure 7:

Jiazhi Yi and Shuyun Wu

Figure 8:

Shuyun Wu

Supplementary Fig 1:

Shuyun Wu

Supplemetary Figs 2-5:

Jiazhi Yi and Shuyun Wu

Supplementary Figs 6-9:

Jiazhi Yi

Signed for and on behalf of the Author(s):	Print Name:	Date:
[illegible]	Bin Wu	2021/11/6
