# Supplementary material for: Berberine alleviates liver fibrosis through inducing ferrous redox to activate ROS-mediated hepatic stellate cells ferroptosis
Source: Cell Death Discov. 2021 Dec 4;7:374. doi: 10.1038/s41420-021-00768-7 (PMC8643357; doi:10.1038/s41420-021-00768-7)
Supplement: Supplementary file 2 — Supplementary table 1 [file 41420_2021_768_MOESM2_ESM.docx]

**Supplementary table 1**

| **Gene** | **Species** | **Primer** | **Sequence** |
| --- | --- | --- | --- |
| ***ATG5*** | Rat | Forward primer  Reverse primer | 5’GCAAGGATGCAGTTGAGGCT 3’  5’ TGGTCCAAAACTGGTCAAATCA 3’ |
| ***ATG7*** | Rat | Forward primer  Reverse primer | 5’ GGCACCCAAAGACATCAAGG 3’  5’ GTGTTGTGCAGGGTTCCCAT 3’ |
| ***COLLA1*** | Rat | Forward primer  Reverse primer | 5’ CATGTTCAGCTTTGTGGACCT 3’  5’ GCAGCTGACTTCAGGGATGT 3’ |
| ***α-SMA*** | Rat | Forward primer  Reverse primer | 5’ CAGGGAGTGATGGTTGGAAT 3’  5’ GATGATGCCGTGTTCTATCG 3’ |
| ***FTL*** | Rat | Forward primer  Reverse primer | 5’TTCGCGGTTAGCTCCATACTCC 3’  5’CGCTTCTCCTCGGCCAATT 3’ |
| ***FTH1*** | Rat | Forward primer  Reverse primer | 5’ TTGCCAAATACTTTCTCCATC 3’  5’ TCCCAGTCATCACGGTCAG 3’ |
| ***ACTB*** | Rat | Forward primer  Reverse primer | 5’ AGAGGGAAATCGTGCGTGAC 3’  5’ CAGGAAGGAAGGCTGGAAGAG 3’ |
| ***ATG5*** | Human | Forward primer  Reverse primer | 5’GCAACTCTGGATGGGATTGC 3’  5’TTGCAGCAGCGAAGTGTTTC 3’ |
| ***ATG7*** | Human | Forward primer  Reverse primer | 5’ GCTCCTTCTGGAGCAGTCAGCCAA 3’  5’ AAGCCCACAGGTCCCCGGATT 3’ |
| ***COLLA1*** | Human | Forward primer  Reverse primer | 5’GTGCTAAAGGTGCCAATGGT 3’  5’CTCCTCGCTTTCCTTCCTCT 3’ |
| ***α-SMA*** | Human | Forward primer  Reverse primer | 5’TTCAATGTCCCAGCCATGTA 3’  5’GAAGGAATAGCCACGCTCAG 3’ |
| ***FTL*** | Human | Forward primer  Reverse primer | 5’ CTTGGGTGTCAAAGGTAAA 3’  5’ ACTGATGCGTGAAGTGCTG 3’ |
| ***FTH1*** | Human | Forward primer  Reverse primer | 5’ ATCTGGCTTGGCGGAATAT 3’  5’ TCAAAGACAACACCTGGGTA 3’ |
| ***ACTB*** | Human | Forward primer  Reverse primer | 5’ GTCTTCCCCTCCATCGTG 3’  5’ AGGGTGAGGATGCCTCTCTT 3’ |
