# Supplementary material for: Berberine alleviates liver fibrosis through inducing ferrous redox to activate ROS-mediated hepatic stellate cells ferroptosis
Source: Cell Death Discov. 2021 Dec 4;7:374. doi: 10.1038/s41420-021-00768-7 (PMC8643357; doi:10.1038/s41420-021-00768-7)
Supplement: Supplementary file 3 — Supplementary Figure legends [file 41420_2021_768_MOESM3_ESM.doc]

**Supplementary Figures**

**Supplementary Fig. 1 BBR alleviates liver fibrosis in mice.**

Mice were divided into: Control group (Ctrl, only i.p. with saline or oil, respectively), Berberine group (BBR, BBR 200 mg/kg/d intragastrically and i.p. with saline or oil, respectively), Liver fibrosis model group (TAA or CCl_4_, TAA/CCl_4_-induced hepatic fibrotic, i.p. with TAA or CCl_4_, respectively), and TAA- or CCl_4_-treated group plus BBR (TAA+BBR or CCl_4_+BBR). **(A, B)** COL4A1, COL1A1 and Desmin were represented in TAA/CCl_4_-induced mouse liver fibrosis by IHC. **(C, D)** The quantification of liver fibrosis with ISHAK score, Masson as well as Sirius red stained in TAA/CCl_4_-induced mouse liver fibrosis. **(E, F)** IHC protein expression rations of Vimentin, Desmin, COL4A1, COL1A1 and α-SMA in TAA/CCl_4_-induced mouse liver fibrosis. Results were expressed as mean ± SD. Data are representative of three independent experiments; n = 6 in every group; Scales: 100 μm, Representative photographs were shown; Compared with the control group, **P*< 0.05, ***P*< 0.01; Compared with the model group, ^#^*P*< 0.05, ^##^*P*< 0.01.

**Supplementary Fig. 2 BBR inhibits HSCs activation *in vivo* and *in vitro*.**

**(A, B)** HSC-T6 and hepatocyte BRL-3A were treated with BBR (50 μM) for the indicated time or different concentration of BBR for 48 h, cell viability was inverstigated by CCK-8. **(C-E)** HSC-T6 and BRL-3A were exposed to BBR (36.8 μM) for 24 h, cell proliferation and viability were investigated by EdU and CCK-8. **(F)** Adopted HSC-T6 with BBR (36.8 μM) for 24 h, whole cell lysate for western blot to investigate Vimentin, PCNA and α-SMA protein. **(G)** Protein expression of Vimentin, α-SMA, and PCNA in CCl_4_-induced mouse liver fibrosis. **(H, I)** Co-staining

of p75^NTR^ with α-SMA in HSC-T6 or in CCl_4_-induced mouse liver fibrosis. Results were expressed as mean ± SD. Data are representative of three independent experiments; n = 3-6 in every group; Scales: 100 μm, Representative photographs were shown; Compared with the control group, **P*< 0.05, ***P*< 0.01; Compared with the model group, ^#^*P*< 0.05, ^##^*P*< 0.01. NS, not significant.

**Supplementary Fig. 3 Ferroptosis contributes to the inhibition of HSCs activation.**

HSC-T6 cells were treated with erastin (10 μM) and sorafenib (10 μM) with or without The indicated inhibitors (Z-VAD-FMK, 10 μM; Fer-1, 1 μM; necrostatin-1, 10 μM) for 24 h. **(A)** Cell viability was assayed by CCK-8. **(B)** Live cells and dead cells were assayed by FDA staining and PI staining, respectively. **(C-F)** ROS generation, cellular Fe^2+^, GSH depletion and lipid peroxidation MDA production were assayed. **(G, H)** Vimentin and α-SMA protein levels were assayed. The results are expressed as the mean ± SD. Data are representative of three independent experiments; n = 3-6 in every group; Scales: 100 μm, representative photographs are shown. Compared with the control group, **P*<0.05, ***P*<0.01; compared with the ferroptosis inducer group, ^#^*P*<0.05, ^##^*P*<0.01.

**Supplementary Fig. 4 Autophagy is involved in BBR-induced depression of HSCs activation in liver fibrosis.**

**(A)** The expression levels of BECN1, p62, LC3B, vimentin and α-SMA were detected by western blot in CCl_4_-induced mouse liver fibrosis. **(B)** Double IF staining of α-SMA and LC3B. **(C)** Western blot revealed that BBR inhibited autophagy in HSC-T6. **(D, E)** Costaining of α-SMA and Atg5 or Atg7. **(F, G)** HSC-T6 cells were treated with or without BBR (36.8 μM) for 24 h, protein expression of Atg5, Atg7, α-SMA, LC3B, and p62 and the relative mRNA levels of Atg5, Atg7, COL1A1 and α-SMA were assayed by western blot and qPCR. **(H)** HSC-T6 cells were treated with or without BBR (36.8 μM) for 24 h, transmission electron microscopy ultrastructural features of autophagosomes are presented. The results are expressed as the mean ± SD. Data are representative of three independent experiments; n = 3-6 in every group; Scales: 1 μm or 100 μm. Representative photographs are shown. Compared with the control group, **P*<0.05, ***P*<0.01; compared with the model group, ^#^*P*<0.05, ^##^*P*<0.01. NS, not significant. NS, not significant.

**Supplementary Fig. 5 BBR inhibited autophagic flux in HSC-LX2.** HSC-LX2 cells were transiently transfected with the GFP-LC3B for 24 h, then cells were treated with vehicle (ddH_2_O), 3-MA (5 mM) or chloroquine (10 μM) with or without BBR (21 μM) for 24 h. **(A, B)** GFP-LC3B puncta number per cell was quantified using the Image-Pro Plus 8.0, the number of the dots were counted in at least three independent visual fields from three independent experiment. **(C, D)** The conversion of LC3B-I to LC3B-II was evaluated by western blot. **(E, F)** HSC-LX2 cells expressing GFP-LC3B were exposed to vehicle, chloroquine (10 μM) or BBR (21 μM) for 24 h, followed by IF assessment by confocal laser microscopy, and p62 puncta per cell were quantified according to GFP-LC3B puncta. **(G, H)** Western blot analysis showed time-dependent p62 accumulation in HSC-LX2 cells incubated with BBR (21 μM) for the indicated time (0, 3, 6, 12, 24 h), as well as the dose-dependent changes (0, 3.125, 21, 50 μM) in the expression of p62 in HSC-LX2 cells incubated with BBR for the indicated time. The results are expressed as the mean ± SD. Data are representative of three independent experiments; n = 3-6 in every group; Scales: 30 μm. Representative photographs are shown. Compared with the control or vehicle group, **P*< 0.05, ***P*< 0.01; Compared with the BBR group, ^#^*P*< 0.05, ^##^*P*< 0.01.

**Supplementary Fig. 6 Suppression of autophagy enhances BBR-**

**induced HSCs ferroptosis.**

**(A-D)** IF staining indicated the expression of FTH1 and Perls'-DAB staining for Ferric ion in both human liver fibrosis and TAA/CCl_4_-induced mouse hepatic fibrosis. **(E)** HSC-T6 cells were treated with BBR (36.8 μM), erastin (10 μM), RSL3 (1μM), sorafenib (10 μM) or specific inhibitors of different cell death pathways (necrostatin-1 10 μM, Fer-1 1 μM, liproxstatin-1 100 nM, DFP 1 μM, Z-VAD-FMK 10 μM and Calpeptin 10 μM). Cell viability was assayed by CCK-8. **(F)** HSC-T6 cells were exposed to BBR (36.8 μM) for 24 h, a whole cell lysate was taken for cellular Fe^2+^, ROS production, MDA generation and GSH depletion investigation. **(G)** HSC-T6 cells were treated with or without BBR (36.8 μM) for 24 h, transmission electron microscopy ultrastructural features of mitochondria. **(H)** Costained α-SMA with GPx4 or Ptgs2 in CCl_4_-induced mouse liver fibrosis. **(I, J)** Co-stained FTH1 or Ptgs2 with LC3B in TAA/CCl_4_-induced mouse liver fibrosis. **(K)** HSC-LX2 and T6 cells were treated with BBR (21 μM and 36.8 μM, respectively) for the indicated time or different concentration of BBR for 24 h, a whole cell lysate was analyzed by western blot using antibodies against the indicated proteins. The results are expressed as the mean ± SD. Data are representative of three independent experiments; n = 3-6 in every group; Scales: 1 μm, 100 μm or 500 nm. Representative photographs are shown. Compared with the control group, **P*<0.05, ***P*<0.01; compared with the BBR group, ^#^*P*<0.05, ^##^*P*<0.01.

**Supplementary Fig. 7 BBR downregulates autophagy to enhance ROS/ferrous-mediated HSCs ferroptosis.**

**(A, B)** IHC for 4-HNE, Perls'-DAB for ferric ion and IF for FTH1 in CCl_4_-induced hepatic fibrosis mouse. **(C)** HSC-T6 was exposed to BBR (36.8 μM), CQ (10 μM) or 3-MA (5 mM) for 24 h, lipid ROS was investigated. **(D)** HSC-T6 was treated with BBR (36.8 μM) or CQ (10 μM) for 24 h, markers of fibrosis, autophagy and ferroptosis were assayed. **(E-H)** HSC-T6 was exposed to BBR (36.8 μM), CQ (10 μM) or 3-MA (5 mM) for 24 h, cellular Fe^2+^, MDA, GSH and cell viability were investigated. **(I)** HSC-T6 were treated with BBR (36.8 μM) with or without CQ (10 μM) or Fer-1 (1 μM) for 24 h, and cell death was quantified by PI staining. **(J-L)** HSC-T6 was exposed to BBR (36.8 μM) followed by trehalose (50 mM) for 12 h, lipid ROS, markers of autophagy, ferroptosis, cellular Fe^2+^, MDA and cell viability were investigated. **(M-O)** HSC-T6 were pretreated with Fer-1 (1 μM) for 1 h, followed by BBR (36.8 μM) for 24 h. lipid ROS, cellular Fe^2+^, GSH, MDA, markers of autophagy and ferroptosis were investigated. **(P, Q)** HSC-T6 was pretreated with DFP (1 µM) for 1 h, following treatment with BBR (36.8 μM) for 24 h, lipid ROS, cellular Fe^2+^, MDA and cell viability were investigated. The results are expressed as the mean ± SD. Data are representative of three independent experiments; n = 3-6 in every group; Scales: 100 μm, Representative photographs are shown. Compared with the control group, **P*<0.05, ***P*<0.01; compared with the BBR group, ^#^*P*<0.05, ^##^*P*<0.01.

**Supplementary Fig. 8 BBR promotes ferritin proteolysis to increase ferrous overload in HSCs.**

**(A)** HSC-T6 cells were treated with BBR (36.8 μM) for 24 h, and FTH1 and FTL mRNA were determined by RT-qPCR. **(B)** HSC-T6 cells were exposed to BBR (36.8 μM) with or without MG-132 (10 μM), protein levels of FTH1, FTL and Ubiquitin were assayed. **(C)** HSC-T6 was exposed to BBR (36.8 μM) with or without cycloheximide (CHX, 100 μM) at the indicated time points, and FTH1 and FTL were assayed. **(D)** HSC-T6 cells were treated with BBR (36.8 μM), CQ or BBR combined with CQ with or without MG-132 (10 μM). FTH1 and FTL were determined. **(E)** HSC-T6 was treated with BBR (36.8 μM) in the presence of MG-132 (10 μM). Ubiquitinated FTH1 and FTL were precipitated and detected with anti-ubiquitin. **(F, G)** HSC-T6 cells were exposed to BBR (36.8 μM) for 24 h, lipid ROS, Fe^2+^, GSH and MDA were assayed. Results were expressed as mean ± SD. The results are expressed as the mean ± SD. Data are representative of three independent experiments; n = 3-6 in every group; Compared with the control group, **P*<0.05, ***P*<0.01; compared with the BBR group, ^#^*P*<0.05, ^##^*P*<0.01. NS, not significant.

**Supplementary Fig. 9 Blockage of ferroptosis offsets the effect of BBR against liver fibrosis.**

**(A, B)** HSC-T6 was pretreated with Fer-1 (1 μM) for 1 h, then the cells were treated with BBR (36.8 μM) for 24 h. and ferroptotic events were investigated. The results are expressed as the mean ± SD. Data are representative of three independent experiments; n = 3-6 in every group; Representative photographs are shown. Compared with the control group, **P*<0.05, ***P*<0.01; compared with the BBR group, ^#^*P*<0.05, ^##^*P*<0.01.
