# Supplementary figures and images for: Berberine alleviates liver fibrosis through inducing ferrous redox to activate ROS-mediated hepatic stellate cells ferroptosis
Source: Cell Death Discov. 2021 Dec 4;7:374. doi: 10.1038/s41420-021-00768-7 (PMC8643357; doi:10.1038/s41420-021-00768-7)

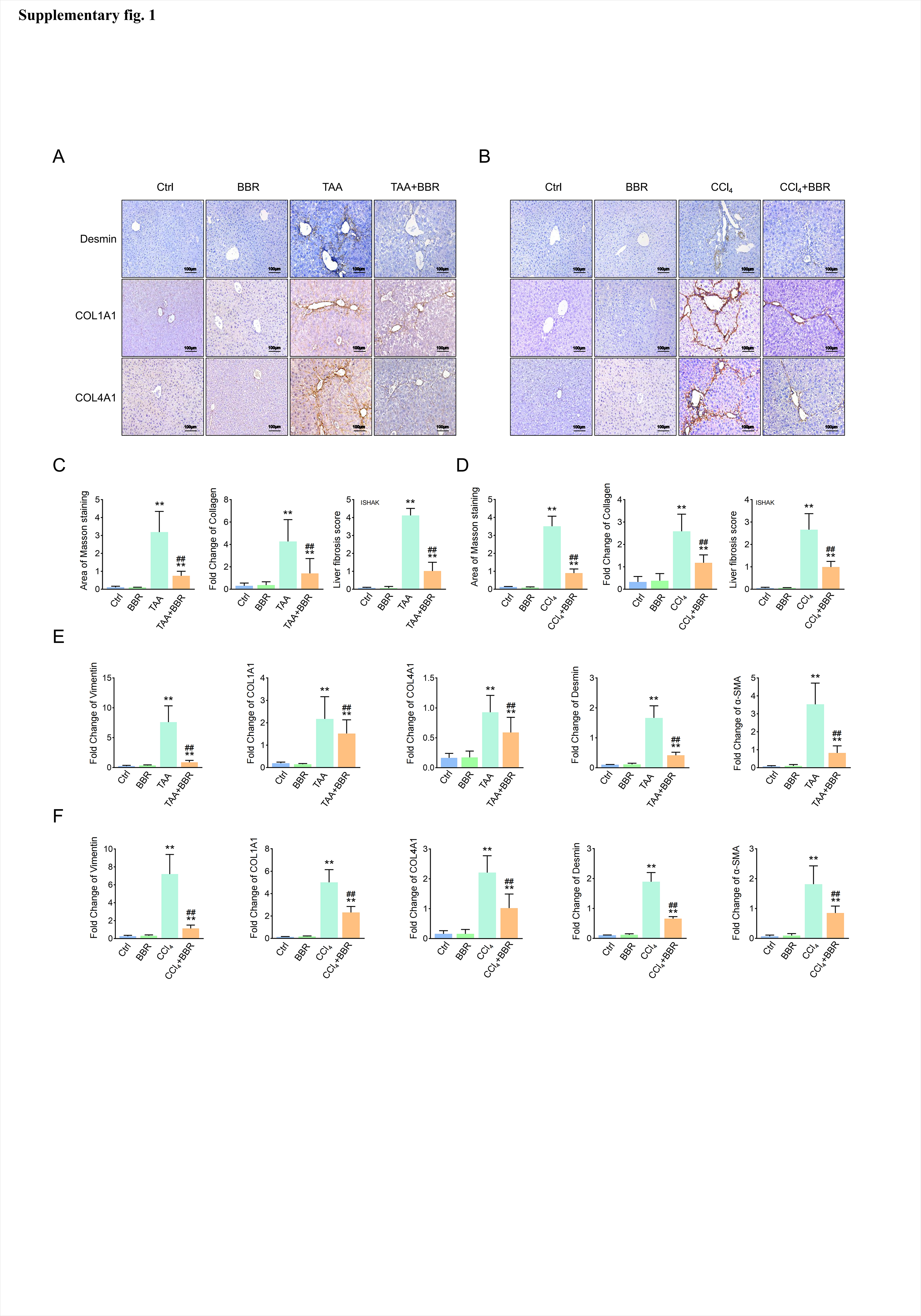

Supplement: Supplementary file 4 — Supplementary Fig. 1 [file 41420_2021_768_MOESM4_ESM.tif]

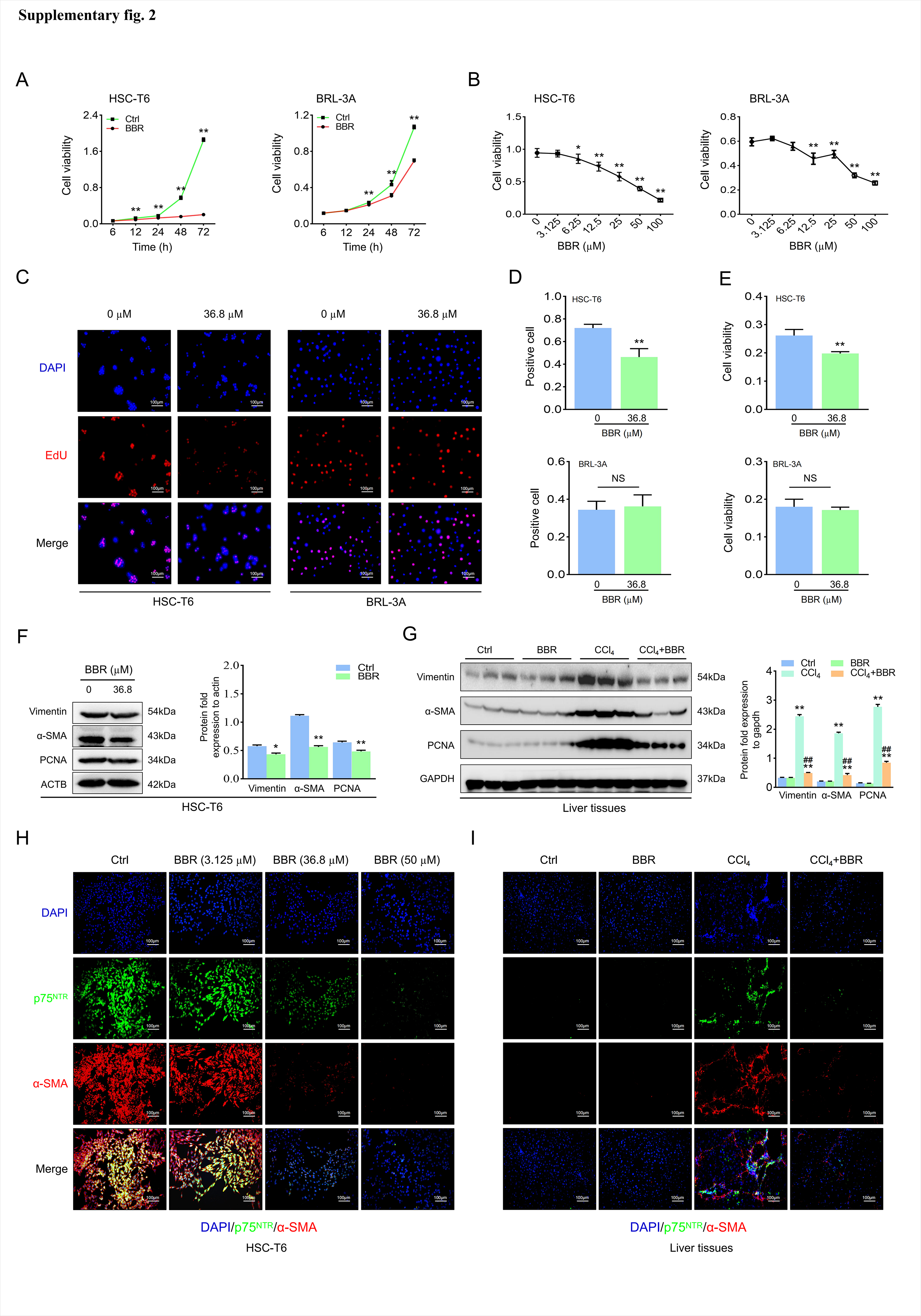

Supplement: Supplementary file 5 — Supplementary Fig. 2 [file 41420_2021_768_MOESM5_ESM.tif]

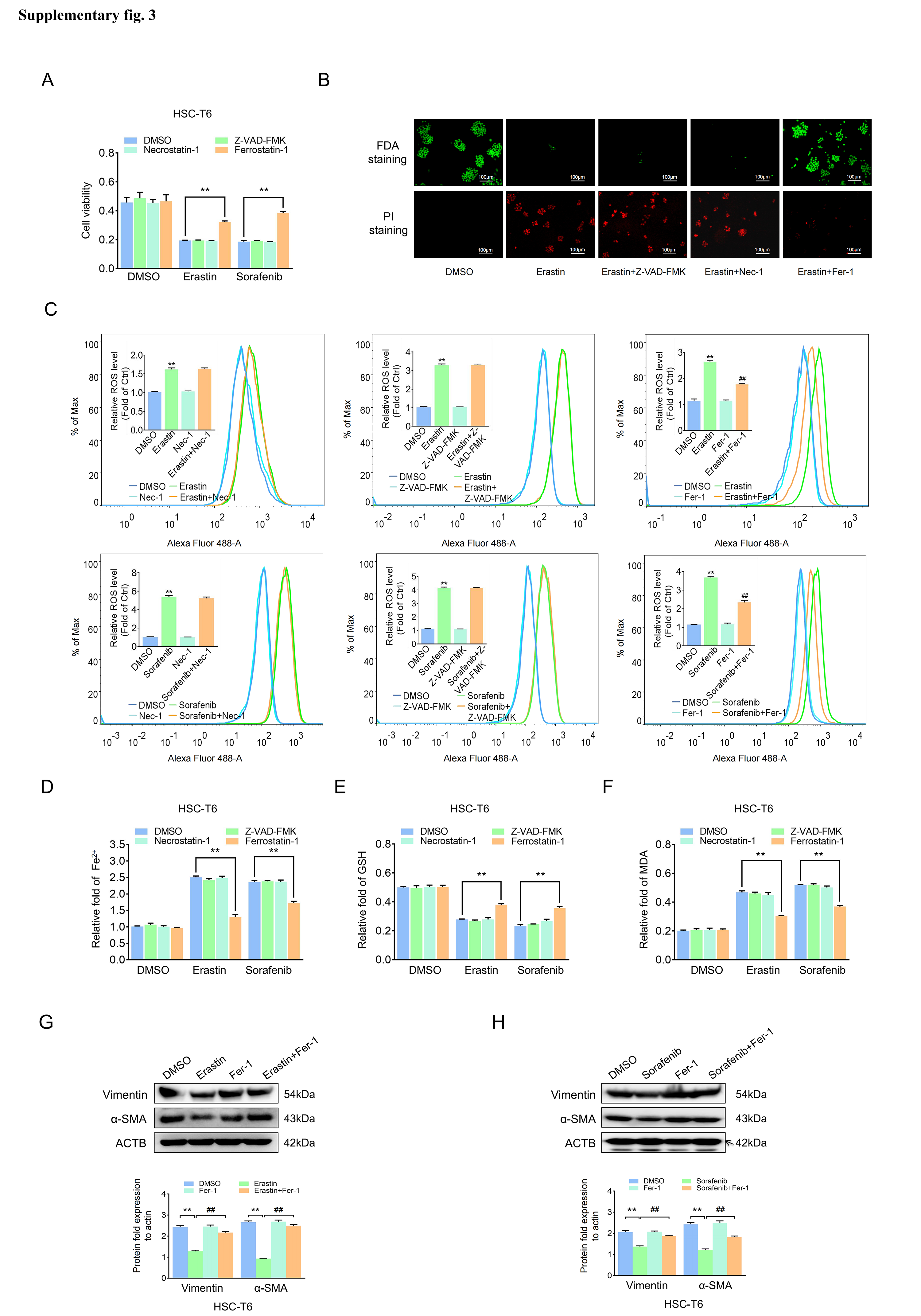

Supplement: Supplementary file 6 — Supplementary Fig. 3 [file 41420_2021_768_MOESM6_ESM.tif]

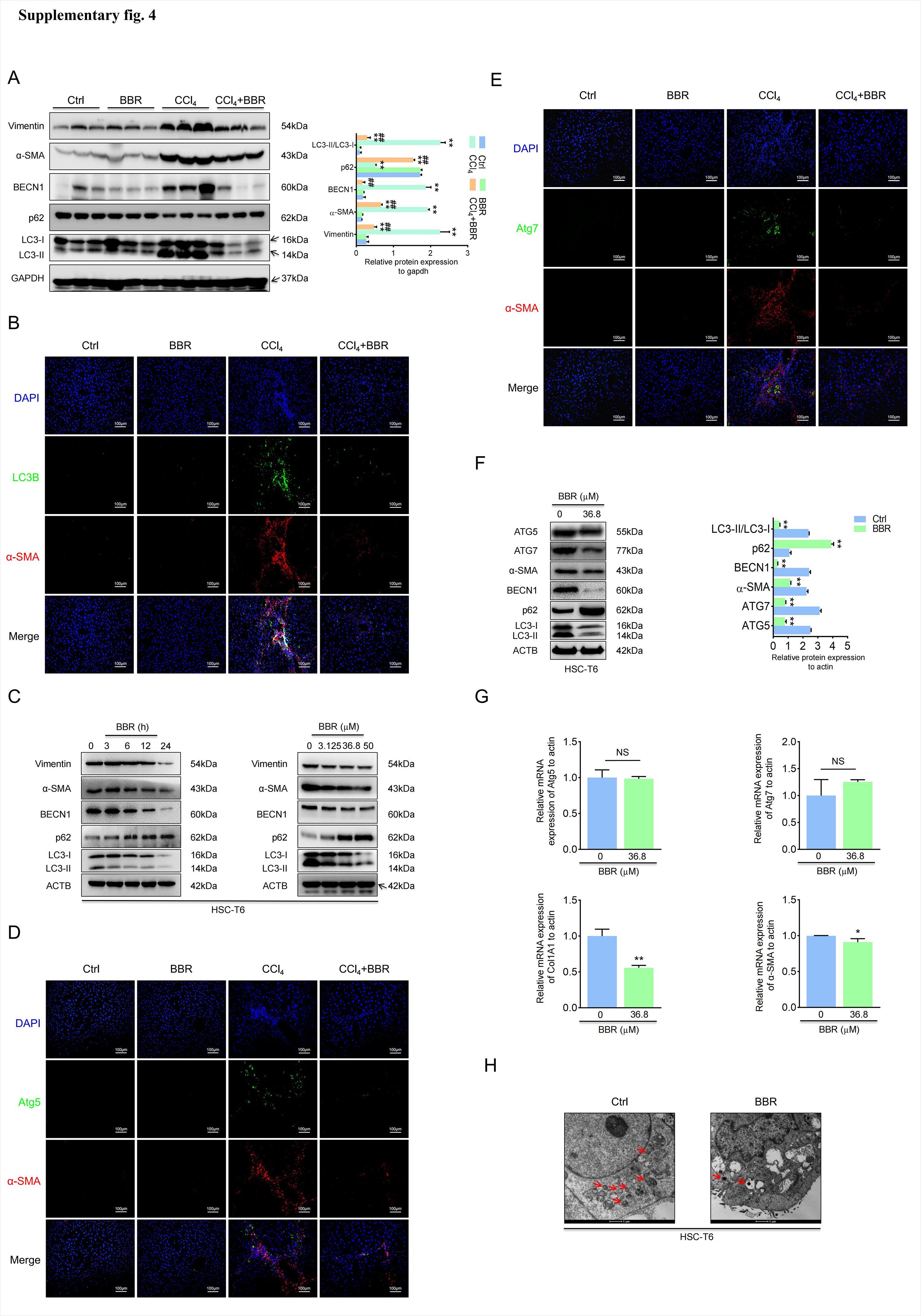

Supplement: Supplementary file 7 — Supplementary Fig. 4 [file 41420_2021_768_MOESM7_ESM.tif]

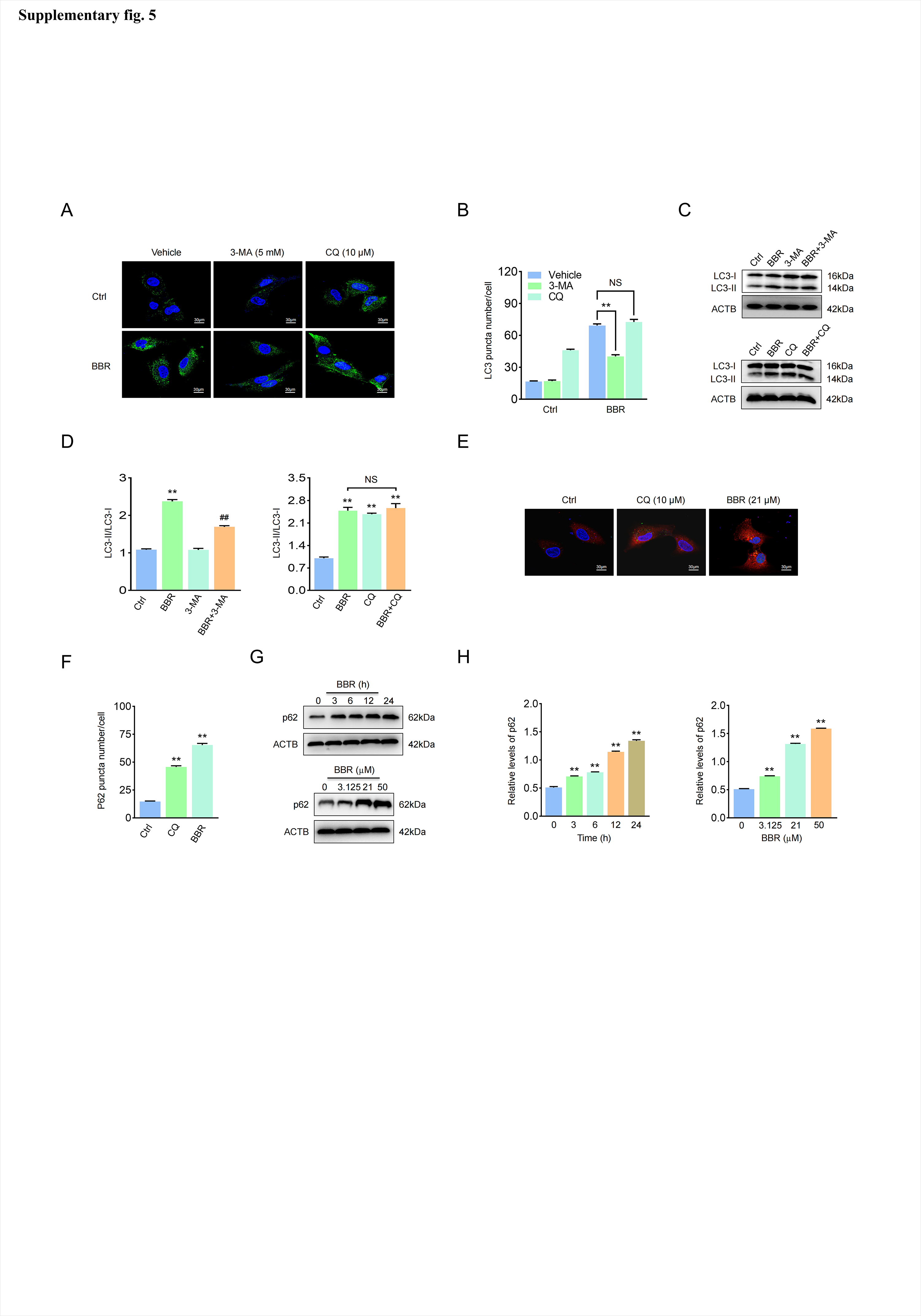

Supplement: Supplementary file 8 — Supplementary Fig. 5 [file 41420_2021_768_MOESM8_ESM.tif]

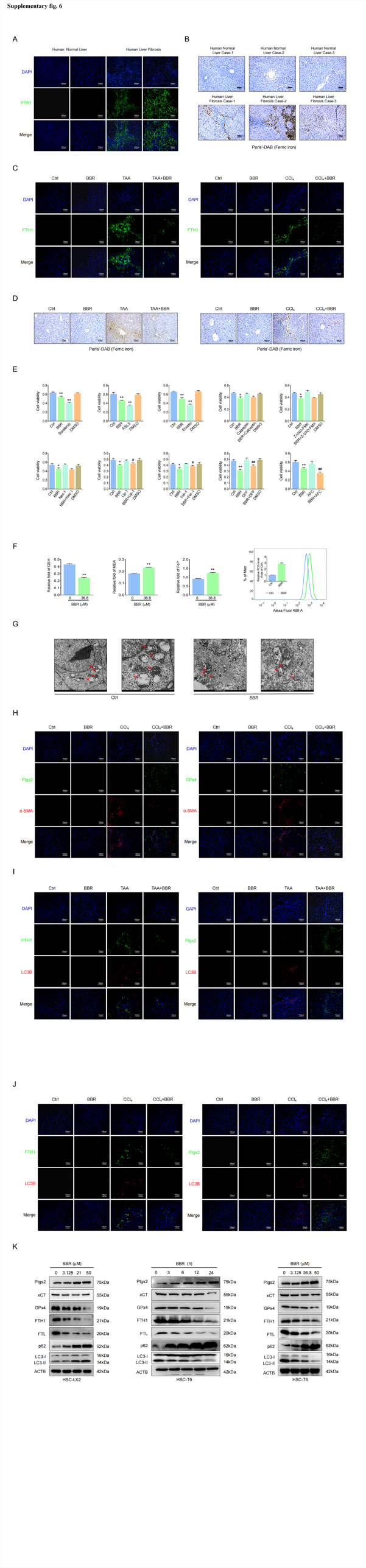

Supplement: Supplementary file 9 — Supplementary Fig. 6 [file 41420_2021_768_MOESM9_ESM.tif]

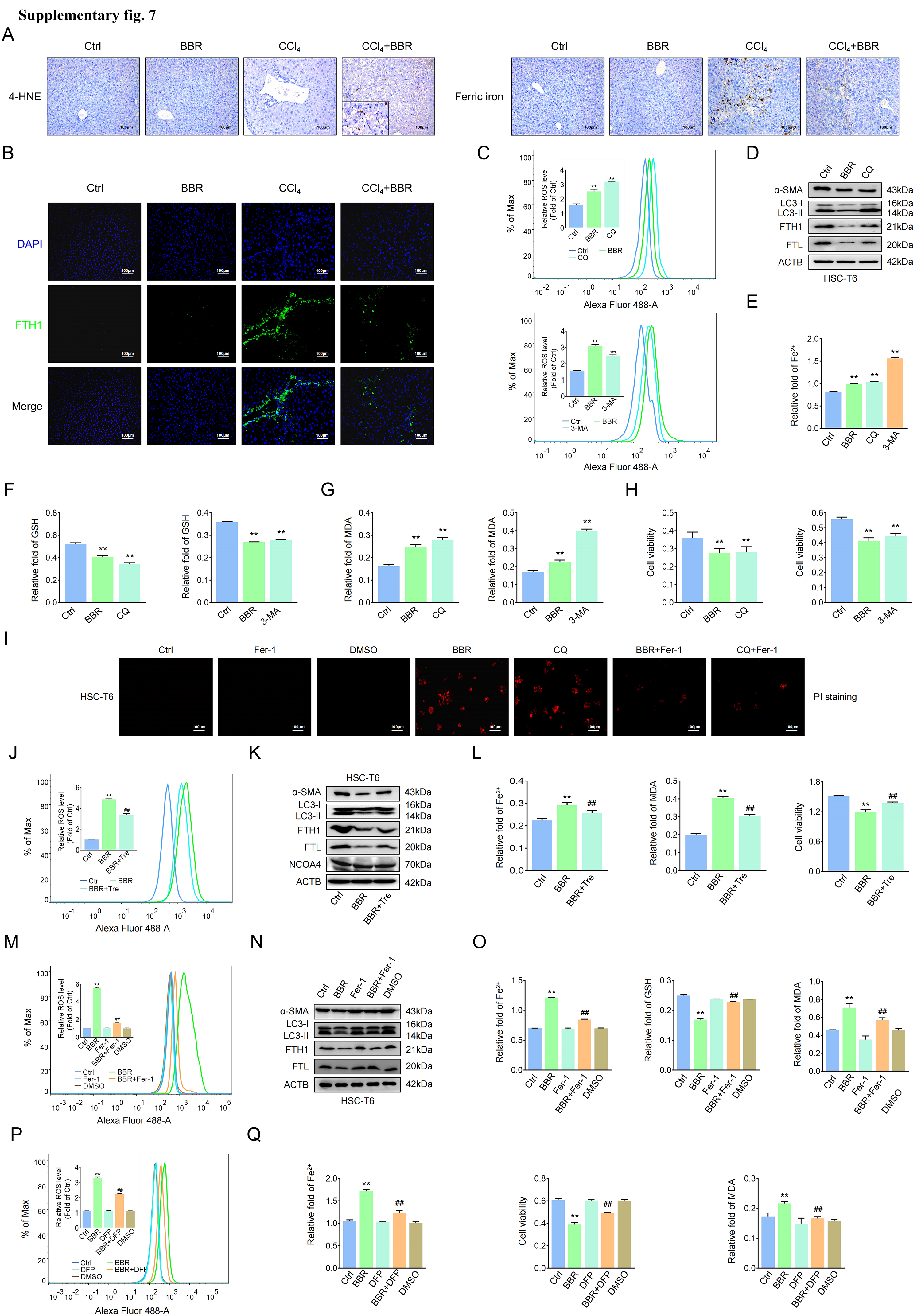

Supplement: Supplementary file 10 — Supplementary Fig. 7 [file 41420_2021_768_MOESM10_ESM.tif]

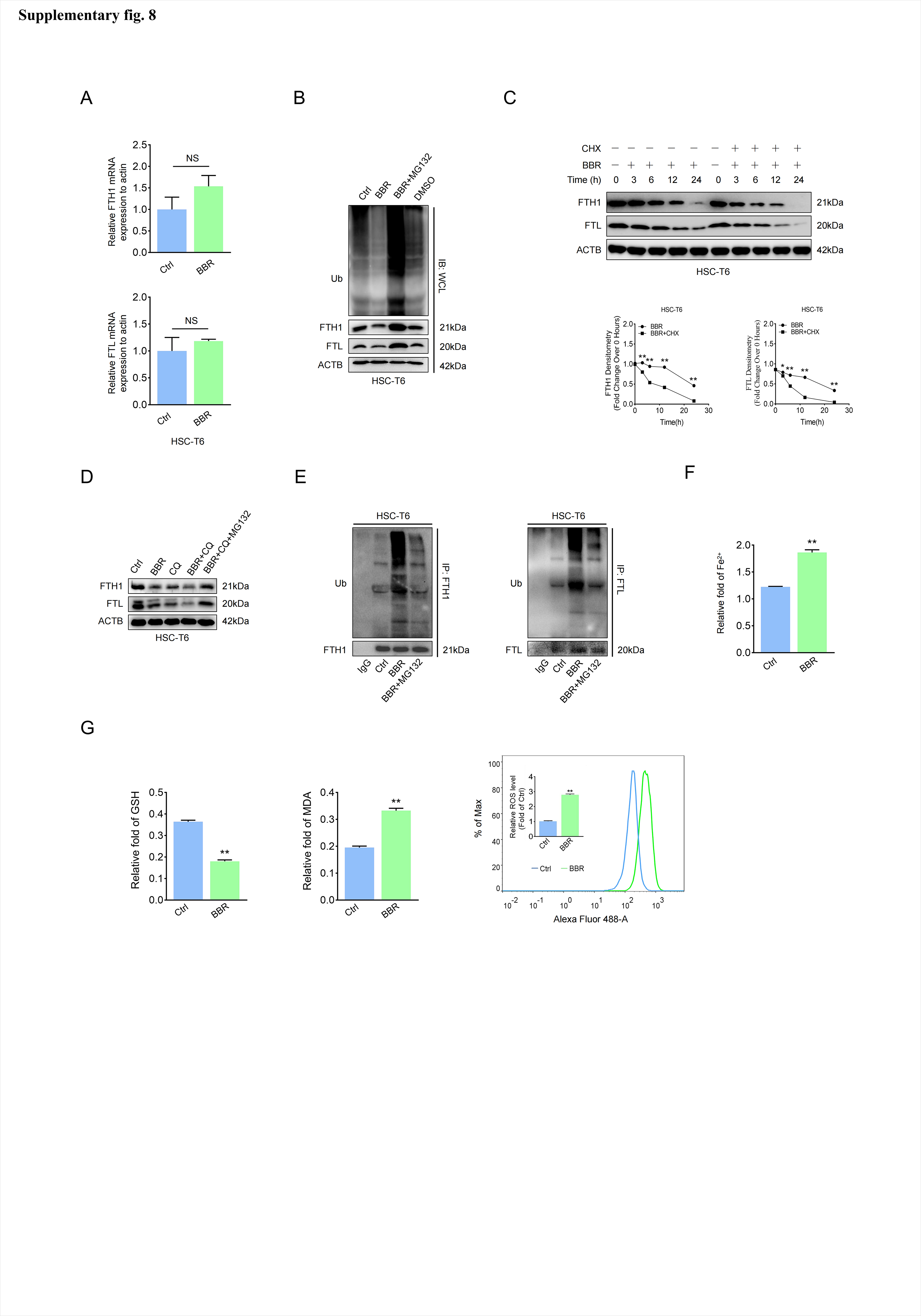

Supplement: Supplementary file 11 — Supplementary Fig. 8 [file 41420_2021_768_MOESM11_ESM.tif]

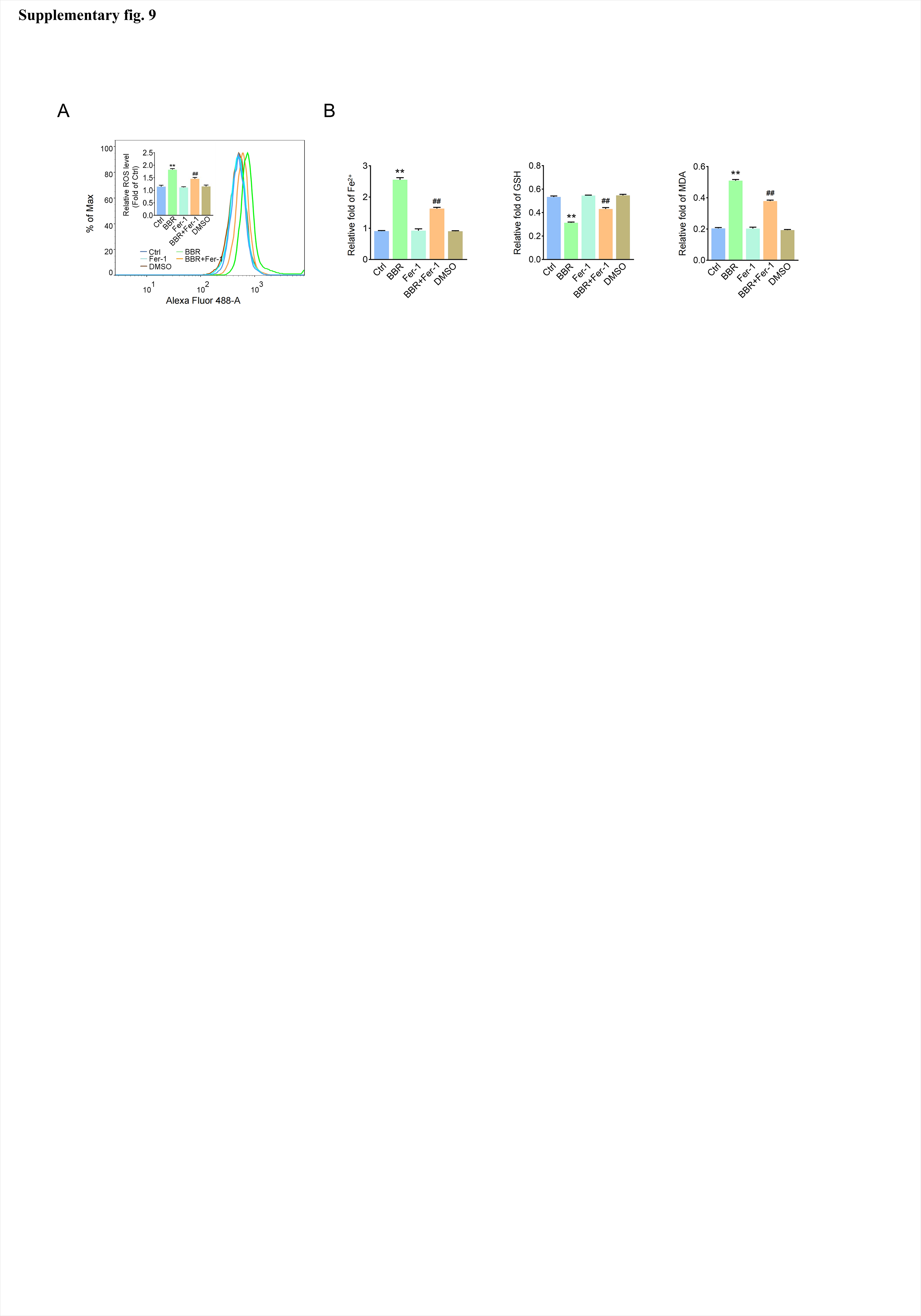

Supplement: Supplementary file 12 — Supplementary Fig. 9 [file 41420_2021_768_MOESM12_ESM.tif]
